# Advances in Nanostructures for Antimicrobial Therapy

**DOI:** 10.3390/ma15072388

**Published:** 2022-03-24

**Authors:** Josef Jampilek, Katarina Kralova

**Affiliations:** 1 Department of Analytical Chemistry, Faculty of Natural Sciences, Comenius University, Ilkovicova 6, 842 15 Bratislava, Slovakia; 2 Department of Chemical Biology, Faculty of Science, Palacky University Olomouc, Slechtitelu 27, 783 71 Olomouc, Czech Republic; 3 Institute of Chemistry, Faculty of Natural Sciences, Comenius University, Ilkovicova 6, 842 15 Bratislava, Slovakia; kata.kralova@gmail.com

**Keywords:** antibiotics, nanoparticles, metals, metalloids, nanoformulations, nanomaterials, polymers

## Abstract

Microbial infections caused by a variety of drug-resistant microorganisms are more common, but there are fewer and fewer approved new antimicrobial chemotherapeutics for systemic administration capable of acting against these resistant infectious pathogens. Formulation innovations of existing drugs are gaining prominence, while the application of nanotechnologies is a useful alternative for improving/increasing the effect of existing antimicrobial drugs. Nanomaterials represent one of the possible strategies to address this unfortunate situation. This review aims to summarize the most current results of nanoformulations of antibiotics and antibacterial active nanomaterials. Nanoformulations of antimicrobial peptides, synergistic combinations of antimicrobial-active agents with nitric oxide donors or combinations of small organic molecules or polymers with metals, metal oxides or metalloids are discussed as well. The mechanisms of actions of selected nanoformulations, including systems with magnetic, photothermal or photodynamic effects, are briefly described.

## 1. Introduction

Various infections are an increasing worldwide threat. Thanks to the introduction of antimicrobial agents, the number of untreatable diseases reduced after the 1950s. The situation changed in the 1980s; since then, morbidity has risen again, and at present, approximately 85% of world mortality from infections is represented by mortality due to respiratory infections, including COVID-19, tuberculosis, and AIDS. The reasons why the number of new infections has increased is general immunosuppression (mainly due to treatment of cancers and the use of immunosuppressive drugs, wide-spectrum antibiotics, and corticoids), a considerable increase in the number of patients with type 2 diabetes, and HIV, and growing resistance to commonly used drugs. During the last decade, nearly 100% increase in the resistance of common pathogens to first-line drugs has occurred. There is also resistance of some strains to second- and third-line drugs [1,2,3,4]; antibacterial chemotherapeutics were divided into several groups according to the broad spectrum of activity, mammal toxicity, means of administration, suitability for “empirical” use from baseline (first choice antibiotics) to critically important antibiotics, the use of which is minimized and allowed in only a few justified cases (see WHO list) [5]. Besides, the development of cross-resistant and multidrug-resistant (MDR) strains is a significant problem [1,2,3,4].

The most common resistant bacterial strains include methicillin-resistant *Staphylococcus aureus* (MRSA), vancomycin-resistant *S. aureus* (VRSA), vancomycin-resistant enterococci (VRE), penicillin-, and macrolides-resistant *Streptococcus pneumoniae*, cotrimoxazol-resistant *Escherichia coli*, the 3rd generation of cephalosporin-resistant *E. coli* and *Klebsiella pneumoniae*, and carbapenem-resistant *E. coli*, *K. pneumoniae*, and *Pseudomonas aeruginosa* [3]. Consequently, important resistance to antimicrobial agents can be found in both Gram-positive and Gram-negative bacteria, causing serious infection [6,7]. For example, MRSA is the predominant agent of nosocomial or healthcare-associated infections affecting 6.5% of all hospitalized patients in the European Union and 3.2% in the United States [8]. Tuberculosis caused by *Mycobacterium tuberculosis* is still one of the most lethal communicable diseases in the world. The spread of multidrug-, extensively drug- and totally drug-resistant tubercular strains is a great problem worldwide [9]. The treatment of infections may be complicated by bacterial resistance, notwithstanding the fact of how mild these infections were initially. The complication can lead to a long-term disorder, treatment failure, or patient death [6,7,10].

The main reason for the selection of resistant microorganisms is the unreasonable application of antimicrobials in human and veterinary medicine [11,12]. In addition, in industrial agriculture and aquaculture, the abuse and overuse of antibiotics accelerates the accumulation of resistant bacteria [13]. Livestock consume almost three-quarters of antimicrobials and unfortunately, drugs are not used only for treatment, but also as prophylactic agents to avert the diseases of animals living in crowded and unsanitary areas; they also serve to support the faster growth of animals and enable animals to digest food more efficiently. This abuse causes the accumulation of antibiotics in the environment, which leads to the contact of pathogens with antibiotics and increases the evolutionary opportunities for the development of antibiotic resistance [14]. Climate change is another cause of the increase in infectious diseases, as warming causes the spread of pathogens and their vectors. Pathogenic organisms get to places where the population, animals, and vegetation are not accustomed to them and do not have centuries-old immunity [15,16,17,18,19,20]. Furthermore, trade in wild animal species that can host dangerous pathogens leads to the transmission of zoonoses [21]. Thus, climate change affects antibiotic resistance on a global scale [22,23].

The most valuable is, of course, the design of structurally new antibacterial agents focused on new (single or multiple) targets [24,25,26,27,28], where one of the basic strategies of drug design is inspiration from natural substances with subsequent modification of model molecules [29,30,31,32]. An interesting approach is the use of modern pesticides as model compounds for the design of structurally new/innovated/modified anti-infectives [24,33], e.g., a strategy to overcome bacterial resistance utilizing metalloantibiotics such as fluoroquinolone-transition metal complexes, was described by Ferreira and Gameiro [34]. In addition to these new entities, the development of so-called chemosensitizers, pathogen virulence inhibitors, bacterial cell membrane disruptors/damagers and the use of combination therapy seem to be a promising strategy against drug resistance [23,24,26,33]. In addition to small molecules, antimicrobial peptides (AMPs) and polymers are also being developed as an interesting alternative to antibiotics [35,36].

The design of new groups of antibacterial agents suitable for further development is becoming more and more complicated, and therefore the discovery of new chemotherapeutics with antibacterial activity carries risks [37]. The strategy of repurposing non-antimicrobial-approved drugs to treat bacterial infections has become popular and is an alternative to reducing risks and accelerating the whole process [38,39]. Another option that does not address the issue of resistance too much is the development of me-too drugs [24]. An alternative to eliminating the undesirable properties of existing drugs, including overcoming resistance, is the application of nanomaterials, to which resistance rarely develops [40,41,42,43,44,45]. For example, nanoformulations for eradication of *Helicobacter pylori* were tested [46], various types of nanoscale carrier systems encapsulating antimicrobials lead to improved efficacy of entrapped drugs against MRSA [47], and nanosystems encapsulating antibiotics, phytoantimicrobial compounds, phages and AMPs have also helped to eradicate biofilms of *P. aeruginosa*, one of the most dangerous biofilm-forming Gram-negative bacteria causing nosocomial and lung infections, as well as catheter-associated urinary tract infections [48]. In addition, naturally occurring AMPs have a very problematic stability and bioavailability, so the use of appropriate nanoscale delivery systems (e.g., metal-based, lipid or polymeric nanoparticles (NPs), and their hybrid systems) can increase the stability of AMPs, ensure their controlled release and targeting, and thus improve their real usability [49,50,51].

The aim of this review article is to summarize the most recent results of nanoformulations of antibiotics and antibacterial active nanomaterials divided according to the materials used and discuss individual nanostructures suitable for their effective encapsulation. Nanoformulations of antimicrobial peptides, synergistic combinations of antimicrobial-active agents with nitric oxide (NO) donors or combinations of small organic molecules or polymers with metals, metal oxides or metalloids are discussed as well. The mechanisms of actions of selected nanoformulations, including systems with magnetic, photothermal or photodynamic effects, are briefly described.

## 2. Nanosystems and Their Benefits

Several colloidal delivery systems such as micro- and nanoemulsions [52], liposomes [53,54,55], solid lipid nanoparticles (SLNPs) [56,57], nanostructured lipid carriers (NLCs) [58,59,60], liquid crystalline NPs [61], biopolymer microgels [62], nanocapsules [63], cyclodextrins (CDs) [64,65,66,67], smart responsive materials polymer-based NPs [68,69,70,71] or dendrimers [72,73] can be used for encapsulation of pharmacologically active compounds. By internalization of drugs to nanocarriers (NCs) their stability, bioavailability, cellular uptake/internalization, and pharmacokinetic profile can be ameliorated along with the reduction of their toxicity [42,74,75,76]. Nanotechnology-based lipid systems, as well as metal/metal oxide NPs showing antibacterial activity, can be successfully applied to control resistant bacteria [42,77,78,79]. Moreover, by encapsulating antimicrobial compounds of natural origin into suitable NCs, “green therapeutics” can be prepared. For example, recent progress and strategies to overcome bacterial resistance by encapsulating phytochemical oils showing antibacterial activity was presented by Gafur et al. [80] The limitations of conventional antibiotics applied in therapies against bacterial infections can be overcome by the use of surface-modified antibacterial NCs able to enhance delivery, bioavailability and effectiveness of encapsulated drugs [81,82]. For example, the use of bacteriophages in the treatment of bacterial infections using nanotechnologies to overcome pharmacological barriers is very interesting [83]. The advantages of using lipid-based NCs, surface modification methods to enhance the efficiency and stability of phage-loaded liposomes, preparation of multiple nanoemulsions suitable for phage cocktails, phage loaded nanofibers; advanced core shell nanofibers enabling immediate, biphasic and delayed release as well as smart phage release delivery platforms were discussed [83,84,85,86]. Bacterial resistance is associated with the overexpression of relative activities of the efflux pump and efflux transporters situated in the membrane of bacteria, which has a crucial impact on the inhibition of the intracellular drug intake and suppression of the drug activities [87,88]. However, the effective inhibition of transporter activity can be achieved using NPs as encapsulating agents, enabling enhanced intracellular accumulation of drugs and helping to overcome bacterial resistance. NPs coupled with natural antimicrobials can be successfully used against MDR bacteria [89]. Besides the penetration of the bacterial cell wall and the destruction permeability of the cell membrane and the structure and function of cell macromolecules due to production of reactive oxygen species (ROS), NPs can kill the bacteria and overcome multi-drug resistance because they are able to affect several targets in bacterial cells and exhibit synergistic effect with conventional antibiotics, resulting in improved antibacterial effectiveness [79,90]. Recent progress in NCs targeting specific bacterial targets and targeting infected cells, which respond to the infection microenvironment and are able to ensure sustained release of antibacterial drugs and their increased levels the site of infection along with minimizing adverse side effects of drugs in non-infected tissues were summarized by Zhang et al. [91]

Targeted drug delivery in NCs is enabled by the use of biocompatible, preferably biodegradable, materials using both passive and active targeting strategies. These mechanisms are primarily controlled by the physicochemical properties of the NCs (composition, particle size, particle shape, zeta potential, specific surface, etc.) [92] Such targeting reduces the burden of infection unaffected tissues. Passive targeting is most often enabled by increased permeation and retention, which depends explicitly on the physicochemical properties of the NC. Furthermore, it is possible to find nanosystems that respond to bacteria by releasing incorporated antimicrobials exclusively in a specific microenvironment produced endogenously by bacteria (locally altered pH, ROS, occurrence of specific (bacterial) enzymes) [93,94]. Stimuli-responsive systems allow the vectorization of drugs to the site of infection. The release of drugs encapsulated in such NCs contributes to an improved antibacterial efficacy, reduced side effects, and microbial resistance [95]. For example, a nanosystem formed from anionic gemini surfactant, chitosan (CS) and vancomycin (VAN) increased antibiotic accumulation at acidic pH and subsequent release in MRSA-infected tissues [96]. Active targeting utilizes specific interactions between the drug carrier and the target cells. NCs are often coated with a variety of ligands that are able to bind to specific receptors expressed on the surface of cells infected with a pathogen or on the surface of bacterial cells, allowing systems to recognize unwanted cells and enter cells via receptor-mediated endocytosis. Coating the NP surface with a biocompatible polymer (e.g., polyethylene glycol (PEG)) prolongs the blood circulation time by preventing opsonization and reducing absorption by the reticuloendothelial system. For example, rifamipicin (RIF) in a mannose and PEG-coated graphene oxide NC is increasingly endocytosed into macrophages via the mannose receptor, thereby increasing the concentration of RIF in macrophages infected with *M. tuberculosis* [97], encapsulated VAN into the pillar [5] arenes covered with mannose are taken up by MRSA-infected macrophages, in which VAN is subsequently released due to the acidic pH and the presence of cathepsin B [98] or NLCs containing ciprofloxacin (CIP) and rolipram coated with retinol to ensure active transport of NLCs via retinol-binding protein 4 to the kidney for the treatment of bacteremia [99]. Dicloxacillin-loaded and CS-coated liposomes have been proven to be a promising NC with increased antibiotic delivery to MRSA [100]. PEG-phosphatidylcholine nanovesicles encapsulating CIP and coated with soyaethyl morphonium ethosulfate (strongly associated with pulmonary surfactant) for pulmonary targeting of extracellular and intracellular MRSA were strongly accumulated in the lungs where CIP was easily taken up by macrophages [101]. VAN-containing NPs coated with the cyclic 9-amino acid peptide CARGGLKSC, identified by phage display on *S. aureus*, increase the accumulation of NPs in *S. aureus*-infected tissues and reduce the required systemic dose, thereby minimizing side effects [102]. In addition, different nanosystems may provide a higher efficacy and lower relapse rates for combination therapy, including the use of photodynamic [103,104] or photothermal [105,106] therapy. For combination therapy, multifunctional nanomaterials are becoming of interest for their effective drug delivery, targeted delivery, and controlled drug release. Subramaniam et al. [107] analyzed the advantages of ‘repurposed’ antibiotics using their encapsulation in micro- or nanosized carrier systems of bioinspired materials, which release antibiotics in response to natural stimuli, enable the transfer of drugs across cellular membranes of infected cells and ameliorate the targeting and specificity compared to conventional antibiotics.

**Figure 1:**
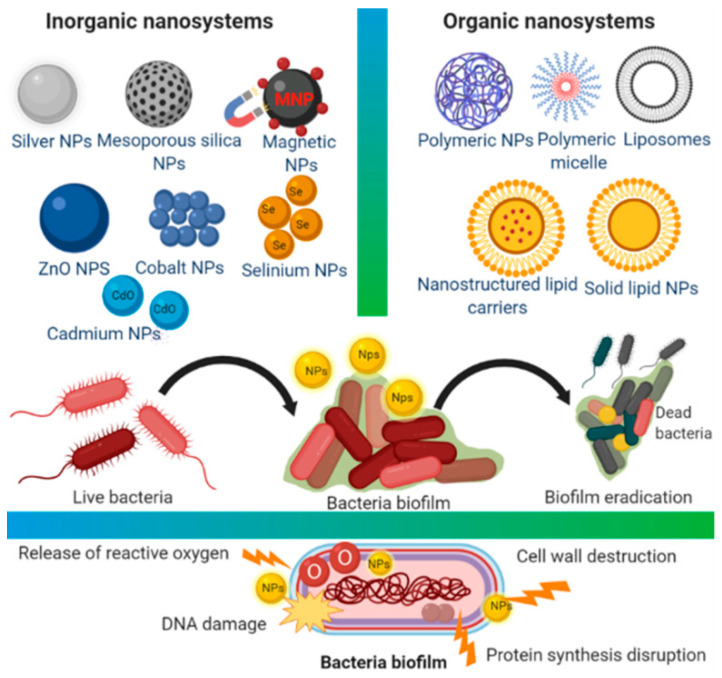
Graphical outline of various classes of nanosystems with illustration of their possible antibacterial/anti-biofilm mechanisms. Adapted from [42], Copyright 2020 MDPI.

Biofilms, i.e., microcolonies of microbes that establish communities with a diversity of microbes, have the same gene composition but various gene expressions, and are usually more virulent than their planktonic counterparts. Biofilm makes bacteria resistant to individuals’ immune systems and conventional treatment [108,109]. For therapies of biofilm-associated skin disorders besides photodynamic therapy application of nanosized formulations including micelles, SLNPs or quatsomes can be used. In addition, ablation of the biofilm matrix can be achieved using NPs producing magnetic, photothermal, or photodynamic effects as a response to external stimuli [110,111,112]. The ability of antibiotic-resistant bacteria on wound surfaces enables their continuous growth, resulting in chronic wound infections and subsequently leading to the morbidity or even to mortality [113]. The role of nanotechnology in combating biofilm-based antibiotic resistance was analyzed by Malaekeh-Nikouei et al. [114] and micro- and nanosystems and biomaterials used for controlled delivery of antimicrobial and anti-biofilm agents contributing to fight-resistant microbes and biofilms were overviewed by Bianchera et al. [115] Organic nanomaterials can effectively reduce the adhesion of biofilms, improve the permeability of antimicrobial agents, or attack the biofilm via specific actions and in such way that they can overcome the problems of bacterial biofilms, which predestine them to be used in the fight against biofilms [116]. Tiwari et al. [117] summarized recent findings related to localized delivery of drugs using medical textiles for treatment of burns and highlighted the benefits of nanofibers with encapsulated drugs showing desirable mechanical integrity and absorption of exudates contributing to acceleration of wound healing. Recently, the development of biologically based natural and synthetic electrospun structures for effective wound healing applications was described [118,119,120]. Colloidal solutions of commercially available metal-based NPs including AgNPs (10 nm and 40 nm), AuNPs (20 nm), PtNPs (4 nm) and ZnO NPs, TiO_2_ NPs, Al_2_O_3_ NPs, Y_2_O_3_ NPs and ZrO_2_ NPs of 100 nm exhibited remarkable antibacterial activity against methicillin-susceptible *S. aureus* (MSSA) and MRSA strains and can be applied as coatings on 3D-printed biodegradable polymers, including bandages for chronic wounds, catheters, etc. [121] Biosafe hydrogels showing a porous structure enabling sustained release of the incorporated antibacterial drugs as well as convenient viscosity, are especially advantageous for topical applications [113]. By using a polymer-based antibiotic delivery system, including polymeric liposomes, polymeric micelles, highly branched polymers and dendrimers, and polymeric nanogels, improved therapeutic effects can be achieved in the treating of bacterial infections compared to free antibiotics [74]. A critical analysis of the use of biopolymer-based aerogels in antibacterial delivery and applications in the wound healing process was published by Yahya et al. [122] Advances in antiseptic formulations and progress in the use of nanotechnology for diagnosing and treating sepsis were summarized by Calle-Moriel and Gonzalez-Rodriguez [123] and Lim et al. [124] Figure 1 shows the various nanosystems and their proposed antibacterial mechanisms of action. 

## 3. Applied Nanomaterials

**Figure 2:**
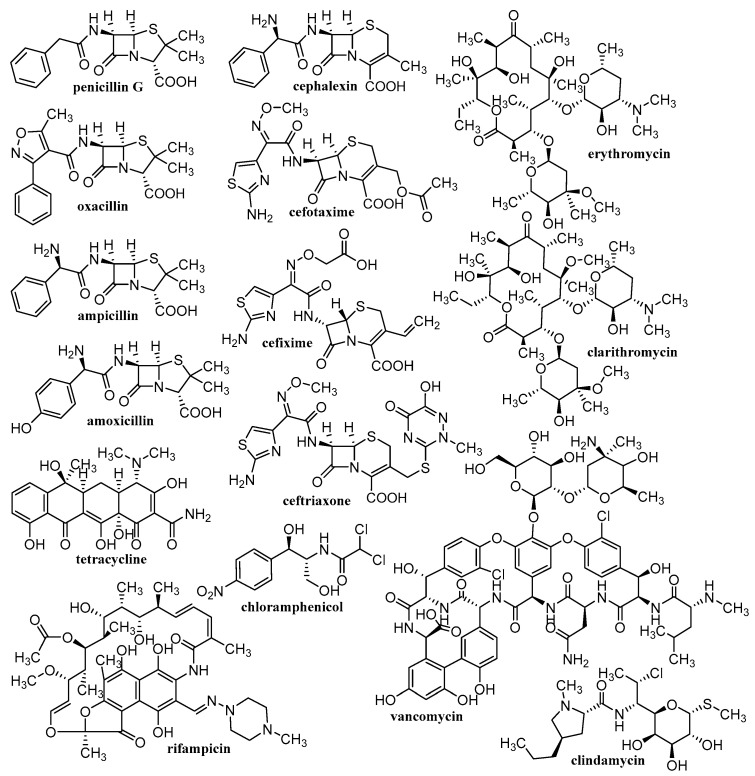
Discussed antibiotics from groups of β-lactams (penicillins, cephalosporins), macrolides, tetracyclines, lincosamides, amphenicoles, glycopeptides, ansamycins.

**Figure 3:**
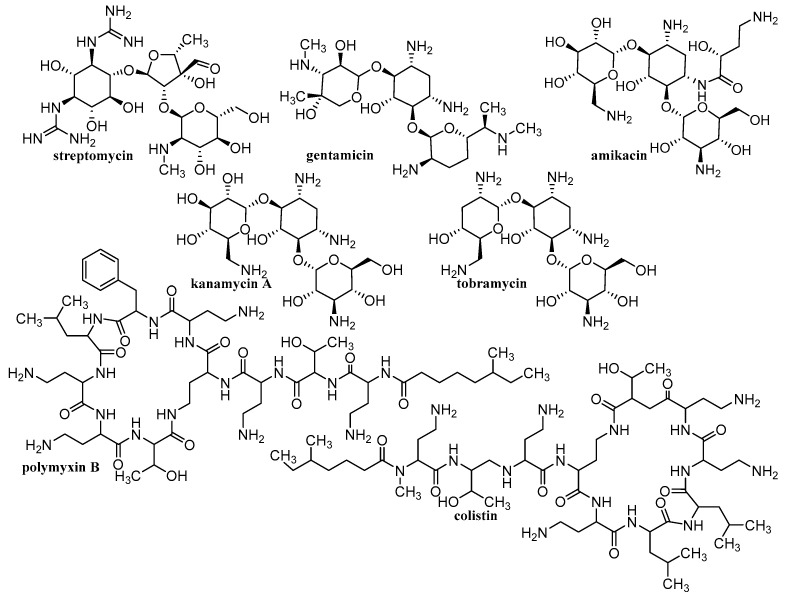
Discussed antibiotics from groups of aminoglycosides and lipopeptides (polymyxins).

**Figure 4:**
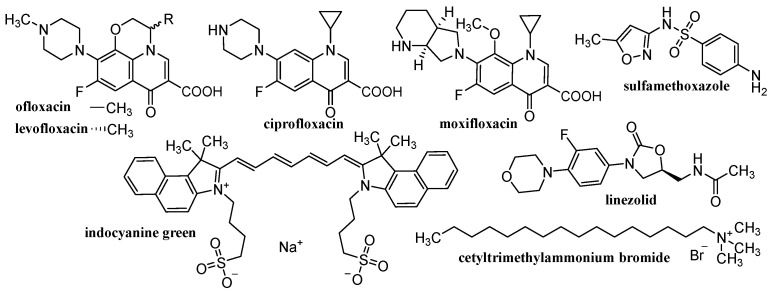
Discussed antibacterial chemotherapeutics from groups of fluoroquinolones, sulfonamides, oxazolidinones and agents from class of disinfectants-antiseptics.

**Figure 5:**
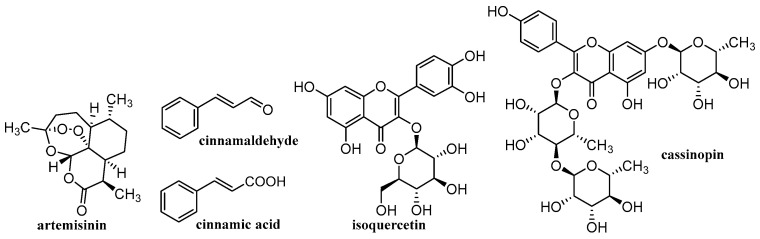
Discussed natural compounds with antibacterial activity.

Various materials are studied for the possibility of creating nanoparticulate carriers. The general classification of these materials is into organic, inorganic and hybrid (organic-inorganic). The advantage of organic materials is their biocompatibility and the possibility of biodegradation to non-toxic products that can be eliminated from the body. These organic materials consist mainly of various polymers, natural (chitosan [125,126,127,128,129,130,131,132,133,134,135], alginate (ALG) [125,126,127,129,130,136], cellulose [125,126,127,129,131,137,138,139], starch [125,127,140,141], gelatin [125,126,127,129,130,142], hyaluronic acid (HA) [132,133], collagen [132,133]) or synthetic, e.g., poly(L-lactic acid) (PLA) [125,126,127,128,129,130,131,132,133,143], poly(D,L-lactic-co-glycolic acid (PLGA) [125,126,127,128,129,130,131,132,133,143], polyvinyl alcohol (PVA) [125,126,127,128,129,130,131,132,133,143] or polymethyl methacrylate [128,132,133,143]). Inorganic materials include metals/metal oxides [128,130,143,144,145,146], silicates/aluminosilicates [144,145,146] and a large family of carbon-based materials [41,45,130,143]. These inorganic carriers are usually functionalized with the above-mentioned organic polymers, resulting in hybrid NCs, in which active molecules are trapped. Depending on the used material, different NCs are formed, such as liposomes/lipid-based delivery systems, polymeric NPs (micelles, spheres, capsules), dendrimers, polymeric complex NPs, CDs, nanocrystals, electrospun nanofibers, electro-sprayed NPs, nano-spray dried particles, covalent organic frameworks, hydrogels, inorganic nanosystems (quantum dots, carbon based NPs) [41,45,52,53,54,55,56,57,58,59,60,61,62,63,64,65,66,67,68,69,70,71,72,73,117,118,119,120,125,126,127,128,129,130,131,132,133,143]. Table 1 provides an overview of the types of nanoformulations discussed and their basic building blocks, while Figure 2, Figure 3, Figure 4 and Figure 5 illustrate the individual anti-infective drugs listed in this review.

### 3.1. Nanostructured Lipid Carriers

NLCs contain both solid and liquid lipids as a core matrix stabilized by surfactants. NLCs that are popular as drug carrier nanosystems due to their biocompatibility, similarly to other lipid NCs such as liposomes, SLNPs, or liquid crystalline nanoparticles (LCNPs) [126,127,128,129,130,131,132,133,134,135,136,137,138,139,140,141,142,143].

Topical delivery of anti-infective drugs using lipid-based NCs such as transfersomes, niosomes, ethosomes, SLNPs, NLCs, microemulsion and nanoemulsion can help to overcome problems associated with poor skin permeation and retention and systematical administration of considerable drug doses. Management of topical infections via topical delivery of antibiotics can overwhelm drug-resistant strains in the skin [147]. Clarithromycin encapsulated in NLCs consisting of glycerol monostearate and oleic acid and poloxamer 188 as stabilizer showing optimized mean particle size of the 164.8 nm, and zeta potential of −39.2 mV exhibited sustained release from the preparation in vitro, while in ex vivo experiment the drug permeation from NLC gel was higher compared to marketed gel (89.5% vs. 65%) because of lipid solubility of NPs in the skin [148]. NLC, with a mean size of 400 ± 14 nm and a zeta potential of −48.9 ± 0.7 mV loaded with clindamycin (CLI) phosphate and RIF when applied on the skin in vitro were found to accumulate into the hair follicle openings, whereby the accumulated amount of CLI did not change in contrast to RIF, the uptake of which increased 12-fold, suggesting that the formulation can be used for the topical treatment of hidradenitis suppurativa [149]. Cinnamon oil-loaded NLC gel formulation showing a mean particle size of 108.48 ± 6.35 nm and a zeta potential of −37.36 ± 4.01 mV exhibited burst drug release for 5 h followed by a sustained release lasting 5 days, and in vitro effectiveness of this formulation against *P. aeruginosa* was confirmed also in an in vivo study, when the treatment lasting 6 days cured the infected burned wound with an improved antibacterial effect [150]. Polymyxin B-coated NLC encapsulating dexamethasone acetate showing a size of 244.73 ± 7.82 nm; and a zeta potential of 2.724 ± 0.458 mV exhibited higher antibacterial activity against *P. aeruginosa* than free polymyxin B [151].

pH-responsive lipid polymer hybrid NPs (LPH NPs) co-loaded with vancomycin and 18β-glycyrrhetinic acid showing a size of 198.4 ± 0.3 nm and a zeta potential of −3.8 ± 0.335 mV exhibited sustained and faster release at acidic conditions and a 16-fold higher antibacterial effect against MRSA in vitro compared to free antibiotics, being able to eliminate 75% of MRSA in less than 12 h [152]. LPH NPs, with a 1,2-dioleoyl-3-trimethylammonium-propane lipid shell and polymeric PLGA core loaded with ampicillin considerably reduced total *Enterococcus faecalis* and boosted the survival rate of protozoa, the concentration 250 μg/mL being the most efficient, and they were effective not only in acute and chronic infections but also in prophylaxis [153]. Ceftriaxone-loaded LPH NPs consisting of CS, glycerol monostearate and polysorbate 80 as a stabilizer exhibited sustained drug release and showed an antibacterial effect against *E. coli* bacteria and were able to reduce the resistance of *Enterococcus faecium* bacteria in patients with cellulitis by 50% [154]. It could be mentioned that, for example, also the nanomedicine-based Moderna and BioNTech/Pfizer vaccines against COVID 19 are based on lipid NPs formulations [155].

#### 3.1.1. Liposomes

As promising nanotechnological strategy to overcome antimicrobial resistance, liposomes [53,54,55] as antibiotic delivery systems can be considered [34]. Liposomes are formed from lipids, self-assembled into bilayers, whereby the liposomal phospholipid bilayer easily fuses with bacterial cell membranes and can release considerable amounts of antimicrobial drugs directly inside bacteria [156]. Antimisiaris et al. [157] summarized findings related to the therapeutic advantages of the localized delivery of liposomal formulations of drugs pre-clinically and clinically investigated in the last 10 years. Therapeutic efficacy of different drugs encapsulated in liposomes in peritoneal dialysis therapy to eliminate bacterial infection in the peritoneal cavity was overviewed by Singh et al. [158] Possible targeting strategies of liposomes against MRSA were summarized by Rani et al. [159] A critical review focused on liposomal delivery systems of antibiotics and non-antibiotic antibacterial agents used for monotherapy and combination therapy against infections caused by *S. aureus* and MRSA was presented by Nwabuife et al. [160] Penicillin G-derived phospholipid NPs increased cellular uptake of penicillin G compared to free drug and effectively eliminated intracellular MRSA in infected lung epithelial A549 cells [161]. CIP encapsulating 1,2-distearoyl-sn-glycerol-3-phosphocholine liposomes ameliorated antibacterial activity of drug and its affinity for bacterial cell surface membrane compared to free CIP and liposome, and reduced the expression level of MepA and NorB efflux pumps of MRSA [162]. The benefits of liposomal drug delivery for the therapy of nontuberculous mycobacterial pulmonary disease and other chronic lung infections were summarized by Chalmers et al. [163] Nebulized liposomal antimicrobials for lung infections can be successfully used for the prevention and treatment of bacterial, mycobacterial and fungal infections [164]. Amikacin liposome inhalation suspension has the potential to be used as an adjunct treatment in the therapy of refractory *Mycobacterium avium* complex lung infection [165].

Efficient treatment of intracellular infection was observed with mannose-decorated liposomes loaded with membrane-impermeable antibiotic gentamicin, which were internalized by both *Salmonella*-infected and non-infected macrophages [166]. Folate-decorated lipid NPs containing VAN exhibited an improved bactericidal effect against MRSA, excellent biofilm inhibition in MRSA as well as increased accumulation in thigh tissues infected with MRSA along with reduced accumulation in the kidney, suggesting that such a formulation can overwhelm constraints of bacterial resistance and negative side effects in kidneys caused by free drug [167]. Pectin-coated liposomes encapsulating amoxicillin did not affect viability of moderately differentiated human gastric adenocarcinoma hyperdiploid cells and mucous-secreting HT29-MTX subclones of HT29-MTX cells up to doses 100 μg/mL but they were cytotoxic against *H. pylori* already at 10 μg/mL. The pectin-coated liposomes, which exhibited mucoadhesion to mucins with a negative charge, adhesiveness to stomach mucin and mucus penetration, and which were able to recognize and adhere to *H. pylori*, can serve as multifunctional drug carriers for local application of antibiotics against *H. pylori* [168].

#### 3.1.2. Solid-Lipid Nanosystems

SLNPs [56,57], similarly to polymer-based nanoscale delivery systems, can improve the absorption and bioavailability of drugs with a low solubility and can protect molecules, which are labile in an acidic environment and can enable the targeting of drugs to their side of action and can reduce adverse side effects [169]; the emergence of antibiotic resistance can be reduced by the incorporation of antibiotics into SLNPs [170]. Ascorbyl tocopherol succinate, which is used as an adjuvant in SLNPs loaded with VAN, showing a mean size of 106.9 ± 1.4 nm and a zeta potential of −16.5 ± 0.93 mV, contributed to a pronounced increase in drug release in an acidified environment compared to controls; as a response to the lipase enzyme, these antibiotic-loaded SLNPs caused double higher growth inhibition of MRSA biofilm for 5 days and a 3.44-fold reduction of bacteria in a skin-infected mice model compared to free drug [171].

#### 3.1.3. Liquid Crystalline Nanoparticles

LCNPs [61] have shown great potential for clinical applications in antimicrobial therapy due to their ability to overcome numerous biological, chemical and physical barriers in bacteria [172]. Pronounced differences in the uptake mechanism of cubosomes (self-assembled lipid NCs of cubic symmetry) into Gram-positive and Gram-negative bacteria are observed. Whereas in Gram-positive bacteria adhering of NCs to the outer peptidoglycan layers is followed by slow internalization into the bacterium, in Gram-negative bacteria, the diffusion of NCs through the inner wall occurs after its fusion with the outer lipid membrane. Rapid internalization of NCs by the Gram-negative bacteria via the fusion uptake mechanism helps to overcome the outer bacterial membrane, ensuring an enhanced toughness to these bacteria, which results in a considerable reduction of the required dose of antibiotics [173]. LCNPs loaded with tobramycin and glycoside hydrolase (PslG), which attacks and degrades the dominant Psl polysaccharide in the exopolymeric substance matrix of *P. aeruginosa* biofilms, protected PslG against proteolysis, showed sustained release of PslG and ameliorated the antimicrobial effect by one till two orders; such NPs enable infection-directed therapy with improved efficiency [172].

Hong et al. [174] investigated the self-assemblies of *E. coli* lipopolysaccharides (LPS) with the human cathelicidin AMP LL-37. AMP LL-37 induced transformation of elongated LPS micelles to multilamellar structures. Whereas treatment of glyceryl monooleate (GMO) cubosomes with LPS activated the swelling of the internal cubic structure, in multilamellar lipid GMO NPs with encapsulated AMP LL-37, it caused transitions into unstructured particles. Hence, antimicrobial materials characterized by an enhanced penetration of LPS layers covering the outer bacterial membrane, resulting in the improved destruction of bacterial membranes, are desirable. The investigation of the effect of the structure, loading and activity of 6 AMPs encapsulated in a lipid-based inverse bicontinuous cubic phase NPs showed that the AMP loading efficiency can be affected by the change of the electrostatic charge and encapsulation improved the antimicrobial activity of AMPs against *S. aureus*, *Bacillus cereus*, *E. coli*, and *P. aeruginosa* [175].

### 3.2. Micelle-like Structures

Cyclodextrin (CD) [64,65,66,67] inclusion complexes, CD coupling, supramolecular hydrogels, and supramolecular micelles are the most frequently used CD-based controlled release systems [176]. By hydrophobic inclusion of oleylamine in β-CD, a supramolecular amphiphile was prepared, forming a self-assembled nanovesicles showing a size of 125.1 ± 8.30 nm and zeta potential of 19.3 ± 9.20 mV exhibiting sustained release of encapsulated VAN during 48 h and showing 2- and 4-fold lower minimum inhibitory concentrations (MICs) against *S. aureus* and MRSA as well as 459-fold reduction of intracellular bacteria using infected human embryotic kidney cells (HEK), and an 8-fold reduction in infected macrophages compared to bare drug [177]. Inclusion complexes of artemisinin and β-CD enhanced the solubility and antibacterial activity against MRSA achieving the inhibition rate of 99.94% after 4 days due to increased membrane permeability and inhibition of respiratory metabolism of MRSA [178]. β-CD gallium NPs prepared using Ga tetraphenylporphyrin and β-CD exhibited sustained drug release for 15 days in vitro and showed synergistic effects with transferrin or lactoferrin against nontuberculosis mycobacteria *Mycobacterium avium* and *Mycobacteroides abscessus* via ROS production and subsequent inhibition of antioxidant enzymes; they exhibited prolonged intracellular inhibitory activity against tested mycobacteria in vitro and their intranasal administration was also found to be effective in a murine lung *M. avium* infection model [179].

Submicrocarriers prepared by electrostatic gelation of anionic β-CD and CS with sizes 400–900 nm and encapsulating CIP using a molar ratio β-CD to CIP of 1:1 were taken up by the macrophage-like cells dTHP-1; although after 2 h incubation the prevailing amount of drug remained adsorbed to the cell surface, and after 24 h incubation the majority of the drug was taken up intracellularly and the subsequent phagocytosis of the carrier ensured its safe degradation and elimination. Such submicrocarriers have the potential to be used as a drug delivery system for the treatment of respiratory extracellular infection with *P. aeruginosa* and/or *S. aureus* [180]. CS NPs based on sulfobutyl-ether-β-CD with sizes 80 and 170 nm and a positive zeta potential loaded with levofloxacin (LEV) exhibited 2-fold higher antibacterial activity against both Gram-positive and Gram-negative bacteria, suggesting their suitability for ocular delivery of the antibiotic to treat ocular infections [181].

Adamantane-capped PEG-poly(ε-caprolactone) (PCL) amphiphilic copolymers linked with β-CD-capped phenylboronic acid-tetraphenylethylene conjugates coupled with ampicillin self-assembling into micelles showed light-triggered and stimuli-responsive release of antibiotics and activation of phenylboronic acid β-lactamase inhibitors, resulting in the destruction of MRSA biofilms via ROS production after illumination and destruction of micelles due to the digestion of PCL segments by bacterial lipase, leading to β-lactamase inhibition. The elimination rate of biofilms was found to be 2-fold higher under illumination compared with β-CD-capped phenylboronic acid-tetraphenylethylene conjugates alone, and the number of live MRSA embedded in biofilms was even 28-fold lower [182].

### 3.3. Polymer-Based Nanosystems

As mentioned above, polymeric nanosystems consist of either natural or synthetically modified or purely synthetic polymers [126,128,129,130,183,184,185]. The potential of amphiphilic polymer therapeutics for their application against antibiotic-resistant bacteria was discussed by Takahashi et al. [186]; the researchers recommended the biomimetic design of synthetic polymers compromising the membrane integrity. Recent progress in responsive polymeric-NPs suitable for treatment against MDR pathogens, which are able to inhibit the formation of biofilms and show improved effectiveness in the eradication of mature biofilms, was overviewed by Su et al. [187]

The bactericidal effects of electrospun polymeric nanofibers are affected by their sizes, diameters and porosity. The surface charge and surface wettability and can be considerably enhanced by incorporation of antimicrobial agents such as metal/metal oxide NPs, carbon nanomaterials, AMPs or natural plant or herbal extracts. The functionalization of electrospun nanofibers with antimicrobial agents can be utilized to fight bacterial infections and resistance and is a promising strategy to combat bacterial infection and resistance [188,189,190,191]. The ethylcellulose/gum tragacanth (85:15) electrospun nanofibrous mats with incorporated honey (5–20 wt%) were evaluated as an effective wound covering biomaterials characterized with antibacterial properties, improved antioxidant activity, good mechanical properties and degradation ability features, and showed proper cell growth, attachment, and proliferation against NIH-3 T3 fibroblast cells [192]. Synthetic AMPs with antimycobacterial activity, HHC-8 and MM-10, encapsulated in PCL-NPs showing sizes of 376.5 ± 14.9 nm and 289.87 ± 17.98 nm, respectively, improved antimycobacterial efficiency of AMPs, resulting in a 4- and 8.33-fold decrease of IC_50_ against *M. smegmatis* and *M. tuberculosis*, respectively, compared to control (75 μg/mL). Moreover, by co-encapsulation of AMPs with RIF, a synergistic effect against *M. smegmatis* was observed due to improved penetration of the bacterial membrane by AMPs, which was protected by encapsulation, enabling the increased accumulation of antibiotics within mycobacteria cells [193].

#### 3.3.1. Chitosan-Based Nanocarriers

CS [125,126,127,128,129,130,131,132,133,134,135], a natural high-molecular-weight linear polycationic heteropolysaccharide that is extracted from shrimp and crab shells and is also found in insect cuticles or in the cell walls of *Zygomycetes*, is characterized with low toxicity [134,135,194]. Positively charged NH_2_ groups of CS, which interact electrostatically with negatively charged groups situated on the surface of bacteria and fungi, increase the permeability of microbial membranes, leading in some cases evenly to cell death. CS in both its bulk and nanoscale form can downregulate the quorum sensing (QS), which is the mechanism used by microbial colonies in a biofilm for modulation and blocking the communication without direct interaction, which results in the eradication of biofilm. Therefore, the use of functionalized CS nanomaterials is effective in combating chronic infections [195]. A review paper discussing pharmacological properties of CS and its derivatives, their mechanism of action against microbes and the factors affecting the antimicrobial activity as well as their activity against resistant bacterial strains, was presented by Confederat et al. [196]

A dual-crosslinked nanocomposite hydrogel fabricated of quaternized CS and CLI-loaded hyperbranched NPs showing good biocompatibility and exhibiting controlled antibiotic release in the acidic environment, was able to kill about 90% of *E. coli*, *S. aureus* and MRSA, suggesting its potential to be used in antibacterial applications [197].

Bacterial biofilms on wounds impair the healing process and often lead to chronic wounds. An NO-releasing CS film designed by Choi et al. [198] considerably ameliorated antibacterial activity against MRSA, reduced bacterial viability by 3 orders and its antibiofilm activity was 3-fold higher than that of the control and CS film. An in vivo treatment of MRSA biofilm-infected wounds with NO-releasing CS film resulted in faster biofilm dispersal, a reduction in wound size, epithelialization rates, and collagen deposition compared to untreated and CS film-treated MRSA biofilm-infected wounds. Amelioration of the complete wound healing process in MRSA-infected wounds was observed using wound dressing of PCL/CS/curcumin (CUR) nanofibers electrosprayed with CUR-loaded CS NPs showing excellent antibacterial, antioxidant, and cell proliferation properties [199].

Spherical AMP LL37-loaded CS NPs fabricated via an ionic gelation method with tripolyphosphate (TPP) crosslinking with a mean size of approximately 127 nm exhibited a considerably ameliorated impact on wound-healing compared to free LL37 [200]. The in vitro estimated MIC value (24 h; pH 7.4) of CS-based hydrogel co-loaded with H_2_O_2_ and AMP against MRSA was approximately half of that estimated for the hydrogel loaded only with H_2_O_2_ or AMP (0.26 vs. 0.53 mg/mL); the sum of the fractional inhibitory concentrations of H_2_O_2_ or AMP of 0.406 suggested a synergistic antibacterial effect against MRSA. The formulation also showed excellent antibiofilm activity in vivo and caused greater wound closure and enhanced wound healing in the in vivo mice models, suggesting that H_2_O_2_-releasing hydrogels have the potential to be used in the successful treatment of chronic wound infection and to eliminate bacterial biofilms without using antibiotics [113]. AMP pexiganan A grafted on CS microspehers (4 μm) exhibited improved bactericidal activity against *H. pylori* J99 compared to free Pexiganan A, even after pre-incubation in simulated gastric conditions with pepsin, via destabilization of *H. pylori* membrane and cytoplasm release; attraction of *H. pylori* to CS promoted the interaction of a grafted peptide with bacterial membrane. Hence, the use of this formulation can present an alternative in the therapy of *H. pylori* [201].

ALG hydrogels based on CS nanocrystals prepared by solid-state aging process and was characterized by a high degree of deacetylation, rod shape and high crystallinity, which showed improved rheological properties and sustained drug release compared to other published CS/ALG systems [202]. Linezolid (LIN) incorporated into both the internal and external surface of the aggregated 3,5-dinitrosalicylic acid-functionalized CS nanosystem with a mean particle size of 150 ± 4 nm showed an antibacterial activity against MRSA resisting to LIN [203]. ALG/CS NPs co-loaded with RIF and ascorbic acid adhered to the bacterial surface, damaged the cell membrane integrity, and were taken up into MRSA cells, resulting in considerable lower MIC values compared with free RIF, and showed potential to be used for treatments of pulmonary intracellular infections [204]. The initial burst release of streptomycin and kanamycin A encapsulated in CS-based gel beads prepared using double ionic co-crosslinking with TPP and ALG during the first three days, exceeding the minimal antibiotic therapeutic concentration of 1–4 μg/mL due to rapid water penetration inside the microsphere, was followed with the sustained release of drugs lasting 11 days and the formulation effectively inhibited the growth of *E. coli* [205].

Adhesive, injectable, conductive, bio-compatible self-healing hydrogel *N*,*O*-carboxymethyl CS with incorporated AgNPs showing antibacterial activity against ATCC and clinical strains of *E. coli*, *K. pneumonia*, *P. aeruginosa*, *S. aureus* and MRSA as well as anti-biofilm activity against *S. aureus*, *E. coli*, and *P. aeruginosa* (ATCC strains) is suitable for the cure of infected wounds [206]. A strong bactericidal effect both in vitro and in vivo against MRSA was also observed with CS–Ag nanocomposites [207] and effective inhibition of MRSA and *P. aeruginosa* biofilms by CS–AgNPs with sizes 10–50 nm using a dose 100 μg/mL was reported by Vijayakumar et al. [208] Nanocomposite of CS-coated AgNPs and nanowires based on graphene showed excellent near infrared (NIR) light-triggered photothermal eradication of *P. aeruginosa* biofilms and the inhibition of bacterial growth [209]. Nanocomposite films consisting of CS, and AgNPs, CuO NPs or TiO_2_ NPs showed superb antibacterial activity against *P. aeruginosa*, *E. coli*, *E. faecalis*, *Streptococcus* sp., *S. aureus* and MRSA and were recommended as a dressing in the therapy of wounds [210]. Antibacterial effectiveness of CS/silicone rubber filled zeolite, Ag and Cu nanocomposites against *P. aeruginosa* and MRSA were reported by Rezazadeh and Kianvash [211]. A nanoscale hybrid consisting of CUR and CS layered on a hexagonal ZnO with average particle sizes of 48 nm showed greater antibacterial activity against MRSA and *E. coli* than antibiotic amoxicillin as well as anticancer activity with 24 h IC_50_ value of 43.53 μg/mL using MCF-7 cell line [212]. Among ZnO, CS–ZnO and ALG–ZnO nanomaterials tested against MRSA, the best antibacterial effectiveness exhibited the nanoscale hybrid ALG–ZnO, showing a low toxicity to the HepG2 cell lines [213]. A CIP-loaded green synthesized CeO_2_/CS nanocomposite showing surface linkage of CS and CIP in CeO_2_ NPs exhibited a higher antibacterial activity against MRSA (MIC: 8 mg/mL) than free drug and can be used for the therapy of MRSA-induced mastitis [214]. CS/PVA hydrogel incorporating 0.5% CeO_2_ NPs biosynthesized using *Zingiber officinale* extract as a reducing and capping agent exhibited higher antibacterial activity against MRSA already after 12 h compared to the control and ensured >90% viabilities of healthy human dermal fibroblasts up to 5 days [215]. MoS_2_ nanoflakes modified with positively charged quaternized CS and loaded with ofloxacin (OFL) adhered to the MRSA membrane and depolarized it by local hyperthermia under NIR irradiation, and at the application of a mild temperature of 45 °C showed superior bactericidal ability. For example, while without laser irradiation the OFL-loaded nanoformulation caused a 2.8 order of magnitude reduction in the bacterial colony, this increased to 5.2 orders of magnitude reduction of bacterial colony after application of NIR irradiation. Moreover, a mild temperature (45 °C) did not damage the neighboring tissue and thus, its co-application with OFL therapy at treating bacterial infections can reduce the development of drug resistance [105].

#### 3.3.2. Alginate-Based Nanocarriers

ALG [125,126,127,129,130,136]/oregano essential oil (EO) composite nanofibers (38–105 nm) containing 2–3 wt% of oregano EO showed ameliorated antibacterial activity against *Listeria monocytogenes*, *K. pneumoniae* and *Salmonella enterica* and pronouncedly increased antibacterial activity against MRSA compared to EO without ALG, suggesting the suitability of this composite to be used in advanced wound dressing technology [216]. ALG–CS NPs encapsulating LysMR-5, an endolysin derived from phage MR-5, with a mean size of 276.5 ± 42 nm, zeta potential of −25 mV, and an entrapment efficiency of 62 ± 3.1% showed a biphasic LysMR-5 release at pH 7.2, cytocompatibility and hemocompatibility and improved bactericidal activity against *S. aureus* [217]. Biocomposite hydrogels based on cellulose nanofibers (CNFs), low methoxy pectin, and Na–ALG with mass ratios of 1:1:1 and 2:0.5:0.5 fabricated using Ca^2+^ ion and citric acid as crosslinking agents with incorporated CLI hydrochloride exhibited prolonged release of the drug, whereby a lower release was observed from the formulation containing a greater CNF portion; the biocomposite hydrogel can be used as a drug delivery system for the therapy of infected wounds [218]. Nisin-loaded pH responsive, mucoadhesive Na-caseinate-Na-ALG coacervate (244 nm; zeta potential of −47 ± 4.31 mV) prevented and eradicated oral biofilm-associated pathogens, e.g., *E. faecium*, *Staphylococcus epidermidis* and *E. faecalis* and such nanoantimicrobials-based coacervate NCs exhibiting their pH-triggered release in the buccal cavity can be recommended to control biofilm-associated oral infections [219]. Efficient delivery of VAN with a subsequent reduction of bacterial colonization and biofilm formation on the implant surface was reported also for transglutaminase cross-linked gelatin-ALG hydrogel encapsulating this antibiotic. This formulation effectively mitigated the implant-related infections in an animal study, which was reflected in a considerably increased bone volume and a more intact bony structure showing only slight inflammatory cell infiltration compared to control [220]. By ionotropic gelation fabricated ALG with poly-L-lysine, which was conjugated with CIP, nanogels were fabricated. The nanogels were stable in dispersion and their films were stable in aqueous environments. However, they were degraded at incubation with trypsin, resulting in antibiotic release due to the presence of enzymatically cleavable peptides with longer lysine sequences carrying an azide function as the end group in the nanogels. By spraying of the dispersion of these enzyme-responsive NPs, implant materials can be coated and the coating can remain stable unless the enzyme is present. However, voluminous groups of the poly-L-lysine linker residue bound to the released antibiotic via the acyl bond impaired its antibacterial activity against *S. aureus* compared to the free drug [221].

Ca–ALG crosslinked phosphorylated polyallylamine encapsulating CLI exhibited sustained drug release, improved cell viability against bone-related human osteosarcoma MG63 cells, and antibacterial activity against MRSA and *Enterobacter cloacae* with MIC values of 275 μg/mL, and 120 μg/mL, respectively, suggesting that the formulation can be used for osteomyelitis affected bone regeneration and rapid recovery of infected parts [222]. PCL–ALG nanofibers mats physically impregnated on the surface with the nanoemulsion-based nanogel of *Mentha longifolia* EO, antibacterial activity of which was tested against of *P. aeruginosa* and *S. aureus*, were able cause ca 80% reduction of *P. aeruginosa* growth at a dose of 5 mg/mL with 1 h exposure and at prolonged exposure of 24 h reduced growth of both standard and clinical strains by 90% was observed. Using a double dose of 10 mg/mL and an exposure period of 3 and 24 h resulted in a practically total growth reduction of tested bacterial strains [223].

Alginate (ALG) hydrogel doped with a NO donor, diethylenetriamine/diazeniumdiolate, was able to release NO over 4 days, and showed strong antibacterial activity against MRSA but only minute toxicity to mouse fibroblasts. This hydrogel exhibited reduced inflammation and rapid wound-size reduction along with improved re-epithelialization, angiogenesis, and collagen deposition, resulting in reduced skin bacterial infection in MRSA-infected wounds in a mouse model [224]. Amikacin and naproxen preloaded hydrogel fabricated via grafting phenylboronic acid to the side chain of the ALG polymer, and showing dual-responsiveness of pH and ROS, exhibited antibacterial and anti-inflammatory properties. In an in vitro experiment amicacin released form hydrogel killed in vitro 90% and 38% of *S. aureus* and *P. aeruginosa*, respectively, while controlled release of naproxen during 24 h under pH 5.0, and 10 mM H_2_O_2_ was observed. In addition, this hydrogel caused 2.8-fold reduction of the levels of pro-inflammatory cytokine TNF-along with 2.41-fold increase of antiinflammatory cytokine IL-10 compared to control hydrogel without amikacin and naproxen and was able greatly diminish the inflammation response of the surrounding tissues and accelerated healing of the infected area [225].

3D-printable nanocomposite ALG-based hydrogel with incorporated bifunctional nanomaterials prepared via functionalization of the pores and outer surfaces of periodic mesoporous organosilica with tetracycline and cell-adhesive poly-D-lysine exhibited pH-dependent release of antibiotic for 7 days and showed considerable antibiofilm activity against *P. aeruginosa*, although it did not reduce the biofilms of *S. aureus* and *E. faecalis* [226]. As a suitable biomaterial for wound dressings showing sustained LEV release and suitable good anti-bacterial activity, LEV-halloysite nanohybrid-loaded fibers based on poly(ethylene oxide) and Na–ALG were reported [227].

Na-ALG/acrylic acid composite hydrogels conjugated to AgNPs as a delivery system of cephalexin was reported by Mohamadinia et al. [228] Photo-crosslinked methacrylated ALG hydrogel with incorporated citrate-stabilized AgNPs (12 nm; zeta potential of −39.9 mV) prevented the direct release of AgNPs, and the bactericidal effect against *E. coli* can be attributed to the release of Ag^+^ ions [229]. Biomimetic injectable double-network hydrogel of oxidized Na-ALG and carbohydrazide-modified methacrylated gelatin with incorporated metal-organic frameworks Au@ZIF-8 was characterized by a considerably ameliorated ROS generation under irradiation with visible light actuation compared to pristine ZIF-8, and exhibited superb bactericidal activity against both *E. coli* and *S. aureus* as well as greatly accelerated wound healing, showing translational potential to be used as a wound dressing material [230].

#### 3.3.3. Starch-Based Nanocarriers

Torres and De-la-Torre [140] summarized the various approaches and techniques used for the modification of starch NPs to optimize the properties needed for successful controlled drug delivery such as chemical modification changing their functional groups, copolymer grafting, as well as physical modification methods (e.g., cold plasma and ultrasound treatment) performed without harmful chemicals. At fabrication of starch NPs, the starch properties, such as amylose content, rheological attribute, morphological characteristics, size distribution, and pasting, are affected [141]. Bioactive and intelligent starch-based films responding to pH-, temperature-, magnetic field-, glucose-, and enzymes can be used not only in food packaging but can also control the delivery of functional ingredients and drugs [231].

By encapsulation of tridoshic rasayana Triphala Churna in starch NPs of 282.9 nm fast drug release at physiological pH 7 was achieved, and the NPs exhibited superb free radical scavenging activity, acetylcholinesterase inhibitory activity and antibacterial activity against *Salmonella typhi* and *Shigella dysenteriae* as well as antibiofilm activity against ATCC MRSA 33591 [232]. After cleavage of the azomethine bond, the conjugate of sodium cefotaxime and potato starch containing 68 mol% of CHO groups enabled prolonged-release delivery of the antibiotic, achieving ~83% after 10 h in normal saline and >90%, after 6–10 h in Tris-HCl buffer, and it was able to maintain therapeutic levels of the antibiotic [233].

Starch-CS anion core polyplexes fabricated via self-assembly of negatively charged starch derivatives and CS derivatives loaded with antibiotic tobramycin (175.2 ± 2.8 nm; zeta potential of −16.8 ± 1.0 mV) and cationic peptide colistin (266.3 ± 6.5 nm; zeta potential of −14.6 ± 0.5 mV) preserved the bactericidal effectiveness of encapsulated drugs against *E. coli* and *P. aeruginosa*, although the blank anion core polyplexes were not active. The molar ratio of carboxyl and amine groups of 10:1 in starch–CS polyplexes was found to be optimal and coating of polyplexes with an additional CS layer enabled the incorporation of enzymes or nucleases (e.g., deoxyribonuclease I) improving penetration of drugs through bacterial biofilms [234].

Green synthesized AgNPs encapsulated in starch showing mean size of 115.2 nm and zeta potential of −17.8 mV exhibited lower cytotoxicity in HEK293 cells but a considerably higher antibacterial activity against *S. aureus* than bare AgNPs [235].

#### 3.3.4. Cellulose-Based Nanocarriers

Cellulose is a biocompatible, biodegradable natural polymer, which can be functionalized, and its functionalized derivatives can be used as wound dressing material, whereby the loading of such dressings with antimicrobials infections in chronic wounds can be controlled, and the effectiveness against drug-resistant bacteria was also observed [137,138,139]. An antibiotic-free biomaterial, probiotic cellulose composite consisting of alive but also metabolically active probiotics *Lactobacillus fermentum* or *Lactobacillus gasseri* within the cellulose matrix showed excellent antibacterial activity against *S. aureus*, *P. aeruginosa* and MRSA and can be used instead of antibiotics for the treatment of topical infections, including severe chronic wounds [236]. Recent progress in the preparation of nanocellulose-based antimicrobial materials containing various functional groups, including aldehyde groups, NH_4_^+^, metal/metaloxide NPs and CS, showing potential to be used as wound dressings and drug carriers, was summarized by Norrrahim et al. [237] The increasing content of aldehyde groups the dialdehyde nanocrystalline cellulose showed superb antibacterial activity against Gram-positive pathogens in vitro and pronouncedly decreased the amount of MRSA on the skin of infected mice models. The low cytotoxicity and superb skin compatibility of this formulation, which does not exhibit acute oral toxicity, predestine it to be used as antibiotic substitute ointments for the treatment of MRSA-infected skin [238].

Bacterial cellulose (BC)–CS NPs composite hydrogels fabricated by γ-irradiation (20–60 kGy) showed superb antibiofilm properties, reflected in up to 90% reduction of viable biofilm and up to 65% reduction of biofilm height and was characterized by a higher porosity, a higher wound fluid absorption and faster in vitro healing compared to respective composite hydrogels prepared with CS polymer [239]. LEV-loaded composite scaffolds of PLA electrospun nanofibrous membranes surface-coated by CNFs and/or silk peptide, in which the CNF coating ameliorated the hydrophilicity and silk peptide coating proliferated conjunctival epithelial cells exhibiting effective bactericidal effects; they were able to stimulate structural and functional restoration of conjunctiva after transplant and thus minimize the post-surgery application of antibiotics [240]. Ampicillin-encapsulated BC/PVA hydrogels ensured 30% of the cumulative antibiotic delivery during 120 h, exhibited superior antibacterial activity against *S. aureus* and *E. coli* and can be used as wound dressings with sustained antibiotic delivery [241]. A biodegradable and biocompatible multifunctional composite hydrogel prepared using PVA-borax gel dual-reinforced with dopamine-grafted oxidized carboxymethyl cellulose and CNFs with incorporated neomycin acting as both an antibacterial agent and a cross-linker, was found to be pH-responsive and it exhibited antibacterial activity against numerous bacteria due to sustained release of neomycin, ensuring a permanent availability of the antibiotic on the wound location [242].

By controlling the loading of AgNPs and living probiotic *Lactobacillus fermentum* at opposite sides of the BC scaffold, the two-sided biomaterial was prepared containing metabolically active probiotics on one surface and AgNPs on the opposite one, whereby the probiotic was protected from the toxic impact of AgNPs. The formulation exhibited improved antibacterial activity against *P. aeruginosa* compared to formulations containing only one of the antibacterials and can be used as an antibiotic-free biomaterial for the treatment of topical bacterial infections [243]. By the incorporation of 0.002% Cu_1_O*_x_* NPs and 0.05% *N*-sulfosuccinoyl-*N*-carboxymethyl CS NPs into cellulose-based hydrogels, it was observed that the rate of wound closure was increased compared to the control (37–39% vs. 65%), resulting in efficient angiogenesis, re-epithelization, collagen deposition in the wound, and antibacterial activity, whereby there was an enhanced NO level in the wound tissue [244]. Flexible polymeric hydrogel films fabricated by integration of Zr-based metal–organic framework UiO-66 with encapsulated tetracyclin in carboxymethyl cellulose matrix, cross-linked by citric acid and plasticized by glycerol exhibited controlled release of antibiotic over 72 h in the artificial sweat and simulated wound exudate media (phosphate-buffered saline, pH 7.4) and considerable antibacterial activity against *S. aureus* and *E. coli*, while it showed a good cytocompatibility towards human skin fibroblast (HFF-1) cells, and can be recommended to be used as antibacterial wound dressing [245]. Biodegradable and biocompatible SeNPs-decorated bacterial cellulose/gelatin composite hydrogel characterized with superior mechanical properties, good swelling ability, antioxidant and anti-inflammatory properties, which exhibited slow and sustainable release of SeNPs, showed excellent antibacterial activity against *E. coli* and *S. aureus* and their MDR counterparts. Moreover, it greatly diminished inflammation, and considerably improved wound closure, granulation tissue formation, collagen deposition, angiogenesis, and fibroblast activation and differentiation in a rat full-thickness defect model, suggesting a superb skin wound healing performance [246].

#### 3.3.5. Hyaluronic Acid-Based Nanocarriers

Spherical self-assembled oleylamine grafted HA [132,133] polymersomes with bilayered morphology encapsulating VAN with sizes 196.1–360.9 nm and a negative zeta potential exhibited sustained drug release for 72 h, and showed a more powerful impact on MRSA membrane and pronouncedly higher antibacterial activity against MRSA than free drug (IC_50_ of 1.9 μg/mL vs 7.8 μg/mL), resulting in higher cell death [247]. Polyelectrolyte complexes of colistin with HA, showing a size of 210–250 nm and a zeta-potential of −19 mV, released 45% and 85% of colistin in 15 and 60 min, respectively, compared to complete (100%) release of drug in 15 min, whereby the antibacterial activity against *P. aeruginosa* (MIC of 1 μg/mL) did not differ from that of the pure drug [248]. The biocompatible composite membrane consisting of biomimetic polydopamine-modified eggshell membrane nanofibers coated with KR-12 AMP and HA showed superb antibacterial activity against *S. aureus*, MRSA and *E. coli*, prevented MRSA biofilm formation on its surface and stimulated the proliferation of keratinocytes and human umbilical vein endothelial cells, increased the secretion of vascular endothelial growth factor (VEGF), and in an in vivo animal study accelerated angiogenesis and re-epithelialization, resulting in rapid wound healing [249].

Using layer-by-layer self-assembly technology, a PLGA multilayer film was prepared with 5 wt% of quaternized chitin as a positively charged constituent and a mixture of fibroblast growth factor 2 and HA (2 wt%) as the negatively charged constituent. Biocompatible preparation with 10 quaternized chitin layers exhibited powerful antibacterial activity against MRSA, *S. aureus* and *E. coli*, and stimulated the proliferation and migration of L929 cells via activation of the cell cycle and epithelial-mesenchymal transformation pathways, while in vivo it promoted wound healing within two weeks via suppressed inflammation, improved collagen deposition, and boosted proliferation and vascularization, suggesting the potential of this formulation as a multifunctional wound dressing material suitable to be used in complex clinical practice. Only antibacterial activities against MRSA were fully evaluated, with a system of 10 quaternized chitin layers showing an inhibition rate of 99.4 ± 10.5% [250]. Self-assembling conjugated oligo(thiophene ethynylene) (OTE)-covalently modified HA (OTE–HA) NPs were found to prevent premature leakage of bactericide, whereas hyaluronidase, largely secreted by MRSA, induced hydrolyzation of OTE–HA NPs, and the release of OTE resulted in the destruction of bacterial cell membranes and subsequent bacterial death; the estimated MIC was 3.3 μg/mL. The OTE-HA NPs showed an effective antibacterial activity against *Streptococcus pneumoniae* and inhibition of bacterial growth was observed even with OTE-HA NPs coated on a surface [251]. NPs of HA-modified zeolitic imidazolate framework-8 (ZIF-8) loaded with VAN were easily uptaken by macrophages, collapsed in the acidic environment and were able to eradicate MRSA with high effectiveness in the macrophage, and suppressed MRSA infections in a mouse pneumonia model [252].

A composite system consisting of a metal ruthenium nanoframe and physically adsorbed natural glucose oxidase coated on the surface with HA, was able to function as the cascade catalyst in the bacterial infection microenvironment by boosting ROS production, resulting in an improved antibacterial activity. This formulation effectively killed bacteria and considerably stimulated wound healing/skin regeneration also in the in vivo experiments, suggesting its potential to be used against antibiotic-resistant bacteria [253].

A review paper focused on HA-based scaffolds loaded with various types of bioactive agents, which can be used in bioactive wound dressings, was presented by Alven and Aderibigbe [254].

### 3.4. Metal and Metalloid-Based Nanomaterials

**Figure 6:**
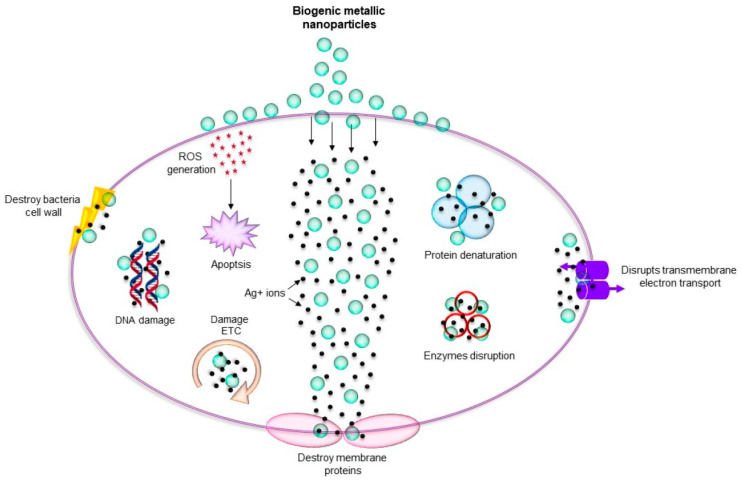
Various mechanisms of antimicrobial activity of metal-based NPs. (ROS = reactive oxygen species). Adapted from [40], Copyright 2018 MDPI.

Metal or metalloid NPs have become extremely popular in the fight against bacterial, viral, fungal and parasitic diseases in both medicine and agriculture [44,125,144,145,146,255,256,257,258,259,260]. While several nano-based preparations can be found on the market among agrochemicals, e.g., [261,262,263], metal and metalloid-based nanosystems are still being extensively tested for medical purposes. Metal or metalloid NPs can be incorporated into polymer chains, as discussed above, or form the basis of a nanosystem that is covered by other materials. It is important to note that a major advantage of metal/metalloid-based NPs used in combination with other antibacterial drugs is the fact that both components potentiate each other and the development of resistance to a given formulation is further significantly reduced. Currently, the so-called green synthesis is very popular, especially for metal-based NP preparations, i.e., their precipitation into a colloidal form using various plant extracts as reducing and capping agents, while the functional groups of active substances from plant extracts coating NP surfaces favorably modify the activity of the formulation. Various mechanisms of antimicrobial activity of metal-based NPs are illustrated in Figure 6.

#### 3.4.1. Silver-Based Nanocarriers

It should be noted at the beginning of this section that by using mass spectrometric approach, Wang et al. [264] identified 38 authentic Ag^+^-binding proteins in *S. aureus* at the whole-cell scale, captured the molecular snapshot on the dynamic action of Ag^+^ against *S. aureus* and found that Ag^+^ can inhibit 6-phosphogluconate dehydrogenase via binding to catalytic His185. Due to multitarget mechanisms, both AgNPs and Ag^+^ ions can contribute to an improved effectiveness of conventional antibiotics and can re-establish the sensitization of MRSA to antibiotics.

AgNPs green synthesized using *Terminalia catappa* leaf extract applied at a dose of 7.8 μg/mL inhibited biofilm formation of MRSA, MDR *P. aeruginosa* and *Candida albicans* by 69.56, 73.7 and 63.63%, respectively, and caused strong damage of the cell wall and membranes of both bacterial strains and *C. albicans*, which is reflected in the considerable loss of membrane and cell wall integrity and profound deterioration of biofilm architecture and the exopolymeric substance matrix, which ultimately resulted in cell death [265]. Colloidal Ag prepared using *Corymbia maculata* aqueous leaf extract, showing particle sizes of 40 mm and 11–16 nm in a colloidal and dried form, respectively, exhibited superior antibacterial activity against plantonic *P. aeruginosa* chronic rhinosinusitis clinical isolates and their biofilms with MIC and minimum biofilm eradication concentration (MBEC) between 0.2 and 3 ppm. On the other hand, the mean MIC and MBEC values of these AgNPs estimated for MRSA, *Haemophilus influenzae* and *Streptococcus pneumoniae*, were in the range of 11 to 44 ppm [266]. Treatment with AgNPs synthesized using *Fraxinus xanthoxyloides* leaf extract applied at a dose of 50 ppm resulted in 81% and 69% inhibition of *P. aeruginosa* and MRSA, respectively, and was able to reduce the *P. aeruginosa* biofilm by 68.6% compared to control [267]. Both chemically and green synthesized AgNPs NPs (3–25 nm) using *Pyrenacantha gandiflora* tuber extracts exhibited excellent antibacterial activities against MDR bacterial pathogens such as MRSA, *K. pneumonia*, and *E. coli* [268]. AgNPs (45 ± 15 nm) biosynthesized using *Dolomiaea costus* extract were reported to kill *E. coli*, *Acinetobacter baumannii*, *S. aureus* and MRSA at a dose of 1 μg/mL [269]. AgNPs prepared using *Agaricus bisporus* basidiomycete mushroom extract as a reducing and capping agent with an average size of 27.45 nm increased the in vitro and in vivo antibacterial activity of VAN against MRSA; in combination with VAN, the AgNPs were found to be effective in the lungs of rats infected with MRSA [270]. AgNPs green-synthesized using cyanobacterium *Phormidium* sp. showed considerable antibacterial activity against MRSA and reinforced the antibacterial activity of chloramphenicol against MRSA; they exhibited a beneficial impact on the wound closure rate, increased the contents of hydroxyproline and antioxidants, attenuated inflammatory cytokines and were able to reduce the epithelization period, thus significantly contributing to wound repairing [271]. AgNPs green synthesized using cyanobacterium *Oscillatoria princeps* exhibited superb antibacterial activities against MDR strains of MRSA, *Streptococcus pyogenes* and *E. coli* reflected in MIC values of 100, 80 and 60 μg/mL [272].

Good antibacterial activity of LEV–AgNPs conjugates against MRSA was reflected in MIC of 10 μM and the conjugates suppressed bacterial adaptive capabilities, resulting in the inhibition of bacterial resistance [273]. Spherical AgNPs (30–36.1 nm) biosynthesized using cellular extracts of endophytic *Fusarium oxysporum* from *Taxus* fauna showed MIC of 100 μg/mL against MRSA and a synergistic antibacterial effect when applied with both VAN and CIP against MRSA, *E. coli* and *P. aeruginosa* [274].

Combined treatment with AgNPs and 400 nm femtosecond laser irradiation (50 mW) showed improved antibacterial efficiency against *P. aeruginosa* and *L. monocytogenes* and MRSA compared to application of AgNPs alone or photoirradiation without application of AgNPs; MRSA was less susceptible to AgNPs and combined treatment than *P. aeruginosa* and *L. monocytogenes* [275].

Cao et al. [276] designed Ag cluster-porphyrin-assembled material consisting of nine-nuclearity Ag_9_ clusters uniformly separated by Ag-centered porphyrin units (AgTPyP), which enabled a long-term charge-transfer states from AgTPyP to adjacent Ag-9 cluster, stimulated ROS production and accelerated the ROS transportation; it was able to kill MRSA and *P. aeruginosa* with an extremely high efficiency (99.99999% and 99.999%, respectively) within 2 h under irradiation with visible light. Moreover, personal masks and protective suits containing this nanomaterials exhibited superior activity against superbugs. Ag/Ta_2_O_5_ nanocomposite showed a superior antibacterial activity against *S. aureus* and *E. coli*, and deposition of its crystallized layer on stainless steel 316 L substrate can be utilized as an adherent, antibacterial layer on the surgical tools to prevent infections on surgical sites [277].

Virus-like mesoporous SiO_2_-coated Ag nanocubes loaded with gentamicin can adsorb on the bacterial cell wall of both *E. coli* and MRSA, and for their superior antibacterial activity against these bacterial strains, small Ag nanospheres produced via intracellular H_2_S in the bacterial environment are responsible. The formulation containing encapsulated antibiotic entrapped in hydrogel dressing completely removed harmful bacteria in diabetic wound and showed the beneficial impact on the wound healing [278]. Hybrids of green-synthesized graphene quantum dots (GRQDs) and AgNPs, in which AgNPs were closely and uniformly surrounded by the GRQDs, exhibited increased effectiveness in MRSA elimination and accelerated the healing of MRSA-infected wounds compared to the application of AgNPs alone [279]. Biodegradable and thermo-responsive antibacterial gelatin methacrylate-based hydrogel containing tannic acid, polyphosphate and gallic acid-functionalized AgNPs can activate the coagulation pathway via released polyphosphate and accelerate the release of tannic acid and Ag^+^ ions due to hyperthermia which originated from Ag NPs, which results in the elimination of 97.57% MRSA and 95.99% of *E. coli* in vitro; its application in vivo killed 91.76% of MRSA in wounds, and improved angiogenesis, and collagen deposition stimulated the wound healing [280]. CS AgNPs decorated with benzodioxane-coupled piperazine showed antibacterial activity against MRSA (MIC: 200 μg/mL) and interfered with the surface adherence of MRSA, suggesting their anti-biofilm activity [281]. Bacteriocin capped AgNPs (16–22 nm) exhibiting low toxicity to normal mice fibroblast 3T3 cells reduced MRSA biofilms to 80–90% because of the ameliorated binding to bacterial cells via small peptides, which occurred on the surface of AgNPs [282].

#### 3.4.2. Gold-Based Nanocarriers

Star-shaped AuNPs with a mean size of 11 nm green synthesized using *Pyrenacantha grandiflora* water extract after conjugation with aceton extracts of *P. grandiflora* showed an MIC of 0.0063 mg/mL against β-lactamase, producing *K. pneumonia* and antibacterial activity of acetone extracts against β-lactamase producing *E. coli* and MRSA was also ameliorated at co-application with chemically fabricated AuNPs [283]. AuNPs (<80 nm) were biosynthesized using a cell-free aqueous extract of *Anabaena spiroides*, which showed antibacterial activity against MDR pathogenic *Klebsiella oxytoca*, MRSA and *S. pyogenes* bacterial strains isolated from clinical samples [284].

AgNPs capped with CS (CS–AgNPs; 21.7 nm, zeta potential of +50.2 mV), glycol CS (GCS–AgNPs; 5.6 nm, zeta potential of +46.5 mV) and poly(γ-glutamic acid) (PGA–AgNPs; 7.4 nm, zeta potential of −37.3 mV) exhibited antibacterial activity, whereby PGA–AgNPs caused higher inhibition of *S. enterica* and *E. coli*-O157:H7 than gentamycin and the antibacterial activity of CS–AgNPs and GCS–AgNPs against tested strains decreased as follows: *L. monocytogenes*, *S. enterica*, *E. coli*-O157:H7, MRSA and *S. aureus*. While attachment of GCS–AgNPs on the surface of MRSA modified the cell, inhibited nutrient flow and disrupted the cell membrane, the PGA–AgNPs penetrated into *S. enterica* and generated cavities, plasmolysis and disintegration [285]. Cinnamaldehyde (CA) attached on the surface of histidine (His)-stabilized Au nanoclusters accelerated ROS production of Au nanoclusters; ligand exchange of surface His–CA with thiols in bacteria was associated with the release of His–CA and consumption of thiols in bacteria, leading ultimately to bacterial cell death. This nanoformulation showed superior antibacterial activity against MRSA and was able to remove the biofilm within 48 h [286]. Multi-layer coated AuNPs fabricated by surface immobilization of AuNPs with polyethylenimine and loaded with antisense oligonucleotides were found to be internalized into MRSA, *S. epidermidis*, and *Bacillus subtilis* cells; MRSA caused silencing of the mecA gene in a dose-dependent manner up to 74% with high selectivity, and in the presence of a β-lactam antibiotic oxacillin bacterial growth, inhibition was ca 71%, suggesting recovery of antibacterial sensitivity [287]. Nanocargos of AuNPs conjugated to ε-polylysine and octadecanethiol (C18) inhibited carbapenem-resistant *Acinetobacter baumannii* and MRSA with 15–20-fold higher efficiency than free ε-polylysine (MIC ranging from 8 to 15 μg/mL) and can be applied for the effective prevention of biofilm formation in both resistant bacterial strains [288].

Topical administration of the ointment of Au/perlite nanocomposite (13–15 nm) green synthesized using *Urtica dioica* extract and its CS-capped derivative promoted healing of wounds, which were infected with MRSA, reduced the healing period and regulated the PI3K/AKT/bFGF signaling pathway, suggesting that this formulation can support the regeneration of MRSA-infected wounds [289]. Nanocomposites fabricated by anchoring AuNPs onto reduced graphene oxide (GO) sheets reduced the biofilm formation in *L. monocytogenes*, MRSA, *E. coli*, *Serratia marcescens* and *P. aeruginosa* by 75%, 78%, 68%, 80% and 79%, respectively. This nanocomposite also caused pronounced inhibition of pre-formed mature biofilms via strong blocking of exopolysaccharides, whereby as a mechanism of antibacterial activity, ROS generation by nanocomposite can be considered [290].

Au nanorods (AuNRs) decorated on the surface with polymethacrylate with pendant carboxyl betaine groups demonstrating a pH-responsive transition from negative charge to positive charge showed improved antimicrobial activity against *E. coli*, *S. aureus* and their drug-resistant strains (MRSA and extended-spectrum β-lactamases producing *E. coli*) compared to AuNRs coated by polymethacrylate with pendant PEG monomethyl ether due to deeper penetration into mature biofilms and showed superior biofilm elimination activities compared to the non-surface charge-transformable formulation [291]. AuNRs decorated on the surface with polymethacrylate copolymers with pendant CIP and the carboxyl betaine groups showed surface charge-switchable activities and lipase triggered on-demand CIP release in sites infected by MRSA and MRSA biofilms, whereby application of photothermal therapy causing disruption of bacterial cell membrane contributed to ameliorated permeability and was sensitivity to antibiotics. Moreover, due to the higher local temperature, the antibiotic release was accelerated, resulting in a further improvement of antibacterial effectiveness [292]. AuNRs disinfect microbes via local heating induced by NIR irradiation. AuNRs conjugated on the surface with cationic AMP LL-37 and neuropeptide ANGIOPEP-2 via electrostatic interactions ameliorated targeting, exhibited photothermal killing of bacteria, and under NIR enhanced cell migration, resulting in better wound healing [106]. It should be noted that superior bactericidal activity against MRSA both in vitro and in vivo also demonstrated a nanocomposite of the AMP BF2b and AuNRs [293]. Nanocapsules consisting of AuNRs coated with pegylated thiol and further modified by the addition of antimicrobial agent CUR and a cell-targeting DNA aptamer, which were exposed to NIR inhibited biofilm formation, caused the death of bacteria via disruption of the bacterial cell wall and membrane due to a photothermal effect. MRSA were captured by these nanocapsules via DNA aptamer targeting, and death of bacteria occurred within 30 min because of the photothermal effect, effective ROS production and loss of transmembrane potential. Resistance against the photothermal treatment was not developed, suggesting the suitability of these nanocapsules for therapeutic applications [294]. The NO-releasing Au nanocages under NIR irradiation showed on-demand quick NO release and generated hyperthermia, which resulted in four orders of magnitude bacterial reduction and 85.4% biofilm elimination in vitro. In an in vivo study using an implant biofilm infection model irradiation with 0.5 W/cm^2^ NIR lasting 5 min caused considerable acceleration of NO release, damaged MRSA biofilms and killed planktonic MRSA missing its biofilm protection [295]. Outstanding synergistic antibacterial ability and cure of MRSA-infected wounds in vivo was observed with 50 μg/mL Au nanoplates combined with 0.1 mM H_2_O_2_ under irradiation with 808 nm laser (1 W/cm^2^) for 3 min [296].

#### 3.4.3. Copper-Based Nanocarriers

CuO NPs (11.4–14.5 nm) fabricated using the leaf extract of *Cymbopogon citratus* suppressed the survival of MRSA, MSSA and *E. coli*, caused morphological deformations of bacterial cells, in which accumulation of Cu was observed; a dose of 2000 μg CuO NPs/mL resulted in the 49.0 ± 3.1% and 33.0 ± 3.2% inhibition of biofilm produced by MRSA and *E. coli* [297]. CuNPs capped with isoquercetin and cassinopin glycosides from *Crotalaria candicans* exhibited >50% reduction in biofilm formation by MRSA, suggesting that for antibiofilm activity the NPs predominantly alter membrane permeability and reduce surface hydrophobicity and the released Cu^2+^ ions are not responsible for this [298]. A lipopeptide produced by human skin bacterium *Paenibacillus thiaminolyticus* and CuNPs/CuO NPs encapsulated in multilamellar liposomes exhibited pronounced reduction of the growth of MRSA and *P. aeruginosa* along with superb antibiofilm activity via their adverse impact on the cell metabolism, secreted virulence such as staphyloxanthin, pyocyanin, and extracellular polysaccharides [299]. Cu clusters prepared using an artificially designed theanine peptide exhibited strong in vitro antibacterial effects against MRSA, *S. aureus, S. epidermidis*, *E. coli* and *P. aeruginosa* due to the destruction of the bacterial wall structure and inhibition of the activity of glutathione reductase associated with ROS outburst, which can result in bacterial death. An in vivo study using Cu clusters revealed their beneficial impact on healing skin wound infections and sepsis caused by MRSA in mice, achieving therapeutic effectiveness comparable with that of mupirocin ointment and VAN along with very low cytotoxicity to normal mammalian cells [300].

Combined treatment with Cu-cysteamine NPs and KI exhibited a considerable antibacterial effect against MRSA and *E. coli* due to the generation of harmful species including H_2_O_2_, $I_{3}^{-}$ and I^−^, ^1^O_2_ and iodine molecules, suggesting the potential of co-application of Cu-cysteamine NPs and KI to be used alone or in combination with antibiotic for the therapy of infectious diseases [301]. Co-application of CuS NPs and indocyanine green, which were activated by NIR irradiation, showed a superior antibacterial activity against *S. aureus* ATCC 29213, *P. aeruginosa* 27853 and clinical MRSA isolates due to combined extra- and intracellular ROS production and it can be supposed that due to excellent tissue penetration, pathogenic microorganisms can be eliminated not only on the skin but also in the soft tissue [103].

An active PCL film with dispersed CuO NPs (0.07% (*w*/*w*)) inhibited growth of MRSA, were found to be hemocompatible, and caused red blood cell breakage <5% [302]. Cu-based 3-D porous nanocomposites containing Cu/CuO NPs, β-CD and reduced GO inactivated MRSA (MIC: 1.93 μg/mL) [303]. Nanocomposites fabricated by the integration of CuS NPs into GO nanosheets showed a needle-like morphology and damaging the bacterial cell membrane physically, which exhibited strong oxidase- and peroxidase-like activity and were found to kill MRSA after the application of a single dose; they can also accelerate healing of infected wounds in vivo [304].

Bimetalic Ag/CuNPs showing positive zeta potential ca. 30 mV at pH 7.2 exhibited strong antibacterial activity against *E. coli* and MRSA due to the prolonged slow release of Ag^+^ and Cu^2+^ ions, being more effective antimicrobials than AgNPs and CuNPs [305]. By incorporation of bimetallic Ag/CuNPs into chemically exfoliated MoS_2_ nanosheets using 5 and 10 wt% Ag/CuNPs to dope MoS_2_, the antibacterial efficiency increased compared to that of Ag/CuNPs [306]. A composite structured cupriferous hollow nanoshell consisting of a hollow Au–Ag core and Cu_2_O acting as photothermal therapeutic agent suitable for the therapy of cutaneous chronic wounds and nonhealing keratitis with drug-resistant bacterial infection was reported by Qiao et al. [307] Whereas Ag released from the hollow AuAg core effectively eradicated MRSA and extended-spectrum β-lactamase *E. coli*, the released Cu^2+^ ions from the Cu_2_O shell supported angiogenesis of the endothelial cell and fibroblast cell migration, which accelerated wound healing. Bone implants incorporating AgNPs and/or CuNPs in the TiO_2_ layer covering the Ti-6Al-4V implant surface released Ag^+^ and Cu^2+^ ions for 4 weeks and produced •OH and •CH_3_ radicals, resulting in strong antibacterial activity in vitro and the implants bifunctionalized with up to 75% AgNPs and 25% CuNPs did not show cytotoxicity and were able to completely eradicate bacteria in an ex vivo murine femora model [308]. Bimetalic CuCo_2_S_4_ NPs exhibiting the conversion of H_2_O_2_ into •OH at a neutral pH due to their peroxidase-like activity showed improved antibacterial activity against MRSA and were able to disrupt MRSA biofilms in vitro and accelerate the cure of MRSA-infected burn wounds in vivo [309]. Bovine serum albumin templated bismuth–Cu_x_S nanocomposites, where Bi is present in the form of Bi_2_S_3_ and bismuth oxysulfides (BZ), showed 14-fold lower MIC against MDR bacterial strains upon NIR irradiation than the composite missing BZ due to reinforced photothermal properties mediated by BiZ/Cu_x_S heterojunctions with surface vacancies and exhibited 90% biofilm inhibition and >75% biofilm eradication. Treatment of MRSA-infected diabetic mice with this formulation under NIR irradiation destroyed the mature biofilm on the wound site, which stimulated collagen synthesis and epithelization and ensured rapid wound healing [310].

#### 3.4.4. Zinc-Based Nanocarriers

ZnO NPs exhibited a strong antibiofilm and potent antimicrobial activity against MRSA, VRSA and linezolid-resistant *S. aureus* isolates recovered from 250 burn wound samples, and they greatly reduced biofilm formation and expression levels of several biofilm genes (icaA, icaD and fnbA) and resistance genes (mecA, vanA and cfr) already at concentrations lower than MIC [311]. Remarkable inhibitory activity of ZnO NPs against VRSA was reflected in zones of inhibition of 10–36 mm and MIC of 625 μg/mL. Exponential reduction in the viability of bacteria was observed with the increasing dose of ZnO NPs and application of 10 mg/mL of ZnO NPs resulted in 73.95 ± 2.17% inhibition of biofilm formation [312]. ZnO NPs of 60 nm showed superb antibacterial activity against *E. coli*, *S. aureus* and MRSA reflected in zones of inhibition of 22 ± 03 mm, 21 ± 02 mm and 17 ± 02 mm, respectively, which was comparable with antibacterial activity of AgNPs of 50 nm [313]. Using ZnO NPs as strong antibacterial and antibiofilm agents against MDR MRSA and their biofilm-associated disease was recommended by Abd El-Hamid et al. [314] ZnO NPs were also reported to prevent MRSA-induced footpad dermatitis in broilers and can be used as dietary supplements [315]. Comparison of the properties of ZnO NPs (17.11–22.56 nm) prepared under different light regimes showed that ZnO NPs synthesized under a green light exhibited the highest free radical quenching and cation radical scavenging activities; those prepared under red, green and blue light exhibited considerable inhibition of amylase, lipase, and urease; ZnO NPs prepared under daylight showed superior total reducing potential and metal-chelating activity; and the most effective antibacterial activity against MRSA was observed with ZnO NPs fabricated under blue light [316].

ZnO NPs biosynthesized using aqueous extract of *Magnolia officinalis* as a reducing and capping agent with the size of 150 nm and zeta potential of +28 mV showed antibacterial activity against MRSA with MIC/MBC values of 250/300 μg/mL [317]. ZnO NPs prepared using root extract of *Raphanus sativus* showing hexagonal wurtzite structure and sizes of 15–25 nm exhibited superior antibacterial activity against *P. aeruginosa* ATCC 27853, MDR *E. coli*, *S. aureus* ATCC 29213, MDR MRSA, *E. coli* ATCC 25922, *E. faecalis* ATCC 29212, MDR *P. aeruginosa*, and MDR *A. baumannii* isolated from diabetic foot ulcers [318].

Se-doped ZnO NPs green synthesized using *Curcuma longa* extract showing polyhedral morphology with a mean size of 40 ± 8 nm and a zeta potential of −28.9 ± 6.42 mV exhibited antibacterial activity against MRSA, with an MIC value of 6.2 μg/mL (MIC value of undoped ZnO NPs was 8.25 μg/mL). Their strong antibacterial activity was associated with enhanced interaction with the bacterial cell wall, increased ROS production, and inhibition of the activity of antioxidant enzymes such as catalase and peroxidase. Moreover, Se-doped ZnO NPs effectively reduced the total protein content of MRSA. However, at the evaluation of oral toxicity and teratogenicity of these ZnO NPs, the renal function test and liver function test in normal and pregnant rats showed moderate changes at the application of a high dose of 2000 mg/kg [319]. Ag-doped ZnO nanostructures green synthesized using *Moringa oleifera* extract with sizes of 54.1 nm and 36.2 nm exhibited 17 mm zone of inhibition against *S. aureus* and effectively inhibited the growth of several fungal pathogens as well [320]. Antibacterial activity of ZnO NPs and Ag-doped ZnO NPs biosynthesized using *Cannabis sativa* extract against *E. coli*, *K. pneumoniae*, MRSA, *P. aeruginosa*, *Salmonella typhi*, and *S. aureus* was reported by Chauhan, et al. [321] AgNPs, ZnO NPs and ZnO–Ag NPs inhibited the rate of MRSA biofilm formation, whereby ZnO–Ag NPs exhibited a synergistic effect against all tested isolates, and suppressed the biofilm formation rate and the icaA gene expression in *S. aureus* strains already at sub-MIC concentrations [322]. A linear low-density polyethylene matrix with TiO_2_/ZnO (1:3) nanocomposites modified bacterial morphology and diminished the bacterial adherence and biofilm formation of MRSA and *K. pneumoniae*, and at a high ZnO weight ratio death of both tested pathogens was observed due to Zn^2+^ release and effective ROS generation [323].

Pancreatin-doped ZnO NPs showed antibacterial, antibiofilm, antimotility and antivirulence properties against MRSA, were able to eradicate MRSA more effective than bare ZnO NPs and pancreatin, and considerably increased the VAN sensitivity of MRSA. They targeted the cell membrane, causing oxidative damage of the cells but were non-toxic to human’s keratinocytes and lung epithelial cell lines at their bactericidal concentration [324]. MIC values against MRSA were observed by coupling of ZnO NPs with chloramphenicol, gentamicin or simultaneous coupling with both antibiotics were 125, 62.5 and 31.125 μg/mL, while MIC observed with bare ZnO NPs was 500 μg/mL, suggesting modulation of MRSA resistance by coupling of ZnO NPs with antibiotics [325]. Potential synergism of ZnO NPs with LEV against the MDR *S. aureus,* including mecA positive MRSA isolates, was reported by Sharif et al. [326] Photosensitizer zinc phthalocyanine (ZnPc) encapsulated in the nanoemulsion containing 5% clove oil and 10%, Pluronic^®^ F-127 and droplet sizes <50 nm showed MIC of 0.065 μg/mL for MRSA and 1.09 μg/mL for *E. faecalis* when its amount in nanoemulsion was 5%. ZnPc-encapsulating nanoemulsion exhibited better photobiological activity than free photosensitizer, although the antimicrobial activity of blank clove oil nanoemulsion was notable as well [327]. Graphene (GR)-based nanoformulation containing CUR and ZnO NPs effectively inhibited the growth of MRSA with MIC of <31.25 μg/mL (MIC values estimated using only CUR or CUR free GR–ZnO NPs were 125 μg/mL); this caused the deterioration of the structure of the bacterial cell wall with subsequent cytoplasm release, resulting in impaired metabolism, and was able strongly to inhibit MRSA topical dermatitis infection in mice as well [328]. A composite drug delivery system prepared via the attachment of carboxylic ZnPc (a broad-spectrum photosensitizer) to UiO-66-NH_2_, which was loaded with LIN and coated on the surface with lysozyme, showed superior antibacterial activity against *S. aureus*, *E. coli*, and even MRSA at irradiation, when ^1^O_2_ was generated, suggesting synergistic antibacterial efficacy of photodynamic therapy and chemotherapy [104].

#### 3.4.5. Iron-Based Nanocarriers

Green synthesized amorphous iron oxide NPs, which were fabricated using *Blepharis maderaspantensis* water extract, showed zeta potential of −20.9 ± 6.24 mV and exhibited strong antibacterial activity against MRSA and *E. coli* suggesting that they can be used for medicinal purposes [329]. Biogenic iron oxide NPs prepared using *Proteus vulgaris* ATCC-29905 (19.23 nm and 30.51 nm; zeta potential of 79.5 mV) exhibited notable antibacterial activity against MRSA; these NPs also were cytotoxic to U87 MG-glioblastoma cancer cells (IC_50_ value of 250 μg/mL) and inhibited the cell migration of the HT-29 cancer cells [330].

Cetyltrimethylammonium bromide (CTAB) loaded in spherical, cubic and tetrapod-shaped iron oxide NPs effectively destructed the robust MRSA bacterial biofilms via active transport of cationic surfactant and magnetic field control. CTAB loading depended on the surface charge density of shapes and not on the surface area of NPs, whereby sharp edges (cubes and tetrapods) ensured improved attachment of CTAB [331]. Manna et al. [332] designed Fe_3_O_4_ NPs functionalized with trisodium salt of *N*-(trimethoxysilylpropyl)ethylenediaminetriacetate and recommended them for applications in magnetic hyperthermia and eradication of MRSA in the presence of an alternating current magnetic field. Extracellular polymeric substances produced by bacteria function as a protective shield preventing penetration of antimicrobials through bacterial biofilms. Magnetic Fe_3_O_4_ NPs can cause considerable mechanical disruption of the matrix, resulting in dispersal of biofilms, and at co-exposure with magnetic fields causing hypertermia, destruction of biofilm matrix can occur. For example, at exposure of MRSA biofilm to 30 mg/mL of functionalized Fe_3_O_4_ NPs of 11 nm to magnetic field the amount of bacteria was reduced by approximately 5 orders [333]. The fourth-generation dendrimer 11, which attached to 48 paracetamol end groups and had 90 units composed of the η^6^-aryl-η^5^-cyclopentadienyliron (II) complex, which was designed by Abd-El-Aziz et al. [334], inhibited bacterial growth of MRSA, VRE and *Staphylococcus warneri*, showing IC_50_ values of 0.52 μM, 1.02 μM, and 0.73 μM, respectively.

Mesoporous iron oxide NPs (MIONPs) and silanized MIONPs with mean sizes <100 nm and superficial area of 258.27 and 186.27 m^2^/g, respectively, which were loaded with CIP, exhibited controlled drug release and better antibiofilm effects against *S. aureus* compared to free antibiotic and drug free NPs [335]. Cinnamic acid-coated magnetic iron oxide and mesoporous SiO_2_ NPs (118–362 nm) loaded with cefixime, sulfamethoxazole and moxifloxacin exhibited higher antibacterial activity against MDR bacteria than free antibiotics; moxifloxacin conjugated to NPs completely eliminated *E. coli* K1 and MRSA, and cefixime-conjugated NPs achieved complete eradication of tested *E. coli* K1 and MRSA bacterial isolates at pronouncedly lower concentrations than the free antibiotic [336]. Rough carbon–iron oxide nanohybrids for NIR-II light-responsive synergistic antibacterial therapy showing a broad spectrum synergistic antibacterial effect against *E. coli*, *S. aureus* and MRSA were designed by Liu et al. [337]; the synergistic antibacterial performances of nanohybrids was also observed in vivo using the rat wound model with MRSA infection. DNA aptamer-conjugated magnetic GO, in which Fe_3_O_4_ NPs were formed on GO, was found to be a biocompatible and light-activated photothermal agent generating considerable local heating under NIR irradiation; at application of NIR irradiation (1.1 W/cm^2^, 808 nm) >97% MRSA cell in aggregated states were inactivated within 200 s [338]. Halloysite nanotubes surface-tuned with Fe_3_O_4_ and ZnO nanostructure showing remarkable antibacterial activity against *E. coli*, *S. aureus* and MRSA, and inhibiting biofilms of *S. aureus* were found to be suitable for therapy of infectious diseases [339].

#### 3.4.6. Titanium-Based Nanocarriers

TiO_2_-NPs biosynthesized using *Ochradenus arabicus* leaf extract were able to reduce biofilm formation of MDR strains of *P. aeruginosa* and MRSA isolated from foot ulcers by 22–70% and caused inhibition of exopolymeric substance production even at concentrations lower than MIC via increased ROS generation [340]. The TiO_2_ NPs (3.4–7.6 nm) showed remarkable antibacterial activity against MDR pathogens and their effectiveness decreased in the following order: MRSA > *E. coli* > *P. aeruginosa*. However, antibacterial activity also depended on the size of TiO_2_ NPs, whereby the highest activity was observed with 4.6 nm and 4.9 nm TiO_2_ NPs and the lowest one with 7.6 nm TiO_2_ NPs; most of the tested TiO_2_ NPs was not genotoxic and mutagenic even at concentration 800 μg/mL [341]. Electrospun TiO_2_ nanofibers that were calcined in a 25% air–75% argon mixture showed excellent antibacterial activities against *P. aeruginosa* and MRSA reflected in MIC/MBC values of 3/6 mg/mL and 6/12 mg/mL, respectively, and they were also found to inhibit bacterial biofilm formation, achieving 75.2% and 72.3% inhibition for MRSA and *P. aeruginosa*, respectively, at treatment with a dose of 2 mg/mL [342].

Ullah et al. [343] investigated the antibacterial activity of erythromycin with TiO_2_ NPs against erythromycin-resistant clinical MRSA isolates and found that the MIC observed for erythromycin using the combination of the antibiotic with 3 mM TiO_2_ NPs (2–16 mg/L) was considerably lower compared to free erythromycin (0.25–1024 mg/L). Ag-doped TiO_2_ NPs green synthesized using *Acacia nilotica* extract showed antibacterial activity against *E. coli*, MRSA and *P. aeruginosa* with the highest effectiveness against *E. coli* and were also cytotoxic to MCF-7 cancer cell lines causing oxidative stress associated with ROS production and lipid peroxidation as well as a reduction of the glutathione level [344]. Porous Ti implants biofunctionalized in the surface using plasma electrolytic oxidation with AgNPs and ZnNPs released Ag^+^ and Zn^2+^ ions from the implant for 4 weeks; implant surfaces with up to 75% AgNPs and 25% ZnNPs completely eradicated both adherent and planktonic MRSA in vitro as well as in an ex vivo experiment performed with murine femora. This solution utilizing a combination of porous design and tailored surface treatment enabled us to reduce the AgNPs amount needed for complete eradication of MRSA by two orders of magnitude and improved metabolic activity of pre-osteoblasts due to the presence of ZnNPs [345]. Polydopamine-ferrocene-functionalized TiO_2_ nanorods showed strong antibacterial activity against MRSA (>95%) and *E. coli* (>92%), which can be enhanced to >99% upon NIR irradiation. Due to the localized hyperthermia and increased production of •OH radicals the functionalized TiO_2_ nanorods inhibited the formation of biofilm as well. Moreover, in the test with pre-osteoblast MC-3T3 E1 cells, they were found to be biocompatible and stimulated cell adhesion and spreading [346].

#### 3.4.7. Selenium-Based Nanocarriers

The observed MIC values of SeNPs against MSSA, MRSA, VRSA, and VRE were 20, 80, 320, and >320 μg/mL, respectively, while the MIC value estimated using Se nanowires (SeNWs) was >320 μg/mL against all tested bacteria. Bactericidal effects of SeNPs against MSSA and MRSA were observed with SeNPs doses 80 and 160 μg/mL, respectively, while application of 40 μg/mL SeNPs exhibited bacteriostatic effects against MSSA. Moreover, SeNPs exhibited a synergistic effect with LIN against MSSA and MRSA via protein degradation [347]. Investigation of the antibacterial activity of SeNPs with sizes ranging from 43 to 205 nm showed that the maximal effectiveness against MSSA and MRSA was observed with 81 nm Se NPs (MIC: 16 ± 7 μg/mL); the SeNPs depleted internal ATP, induced ROS generation, and disrupted the membrane potential. It was stated that already a dose of 10 μg/mL of 81 nm SeNPs can kill *S. aureus* without damaging mammalian cells [348]. SeNPs of 30–70 nm exhibited in vitro antibacterial activity against MRSA and methicillin-resistant *S. epidermidis* already at a dose of 0.5 ppm. When these SeNPs were applied as a coating via surface-induced nucleation-deposition on Ti implants and tested in an infected femur model in rats, they exhibited a powerful inhibition of biofilm formation on the implants and diminished the amount of viable bacteria in the surrounding tissue, suggesting that SeNPs coatings can help to reduce antibiotic-resistant orthopedic implant infections [349].

However, the furcellaran (hybrid β/κ-carrageenan) films modified by SeNPs showed antibacterial activity against *S. aureus*, MRSA and *E. coli* reflected in the inhibition zone diameters of 21.8 mm, 26.6 mm and 26.7 mm, respectively [350], and similar results were observed with furcellaran-gelatin films with Se–AgNPs [351]. A nanosystem consisting of Ru–Se NPs, a natural red blood cell membrane and gelatin NPs, able to release Ru-Se NPs after degradation of gelatin NPs by gelatinase, destroyed the bacteria cells, showed superb antibacterial activity in vivo in an MRSA-infected mice model and ameliorated the wound healing [352].

#### 3.4.8. Silica-Based Nanocarriers

SiO_2_-gentamicin nanohybrids exhibited antibacterial activity against MRSA (MIC: 500 μg/mL) and completely eradicated *E. coli* cells in biofilms at 250 μg/mL, causing wrinkling of bacterial cell walls and deterioration of the shapes [353]. Nanostructured Ag@SiO_2_–penicillin NPs containing 33.2 wt% of triangular Ag nanoplates and ca 2.8 wt% of grafted antibiotics showed a strong bactericidal effect against MSSA and MRSA bacteria and showed no cytotoxicity against A431 cell line [354]. Using Au–SiO_2_ core-shell mesoporous NPs loaded with amoxicillin a 20-fold reduction of the amounts of antibiotic needed to treat β-lactam resistant *P. aeruginosa* and MRSA was observed, which in the case of MRSA corresponded to the reversion of resistance [355]. 80SiO_2_–15CaO–5P_2_O_5_ mesoporous bioactive glass loaded with AgNPs (<5 nm; mole ratio in the range 1–10) exhibited the same in vitro antibacterial activity against MRSA with MIC of 10 mg/mL, independently of the loaded AgNPs amounts [356]. Mesoporous SiO_2_ supported Ag–Bi NPs exposed to laser irradiation remarkably discarded the mature MRSA biofilm and reduced 69.5% of its biomass at a dose 100 μg/mL in vitro. Moreover, the formulation was able to kill ca 95.4% of bacteria in abscess and accelerated abscess ablation in vivo, showing potential to be used in treatment of skin infections [357]. The bactericidal rate of core–shell-structured silicon-based NiOOH nanoflowers applied at a dose of 200 mg/mL to *P. aeruginosa*, *K. pneumonia* and MRSA achieved 99.9%, which can be associated with their high surface area and the high oxidative effectiveness of Ni^3+^ ions existing on its surface; the cytotoxicity of the formulation towards mouse embryonic fibroblasts was inappreciable [358]. Glycol CS and polydopamine grafted Cu-SiO_2_ NPs exposed to NIR light released Cu ions and exhibited rapid and long-term inhibition of MRSA *E. coli* and biofilms. Moreover, due to “mild hot ions effect” of the formulation migration and angiogenesis of endothelial cells is promoted and macrophages polarization into a pro-inflammatory M1 phenotype can occur enabling suppression of the infection via the immune–antibacterial effect; the formulation under laser irradiation was also effective in vivo in wound healing process [359].

## 4. Conclusions

Microbial infections caused by a variety of drug-resistant and MDR microorganisms are becoming more common. In contrast to this unfortunate trend, there are fewer and fewer approved new antimicrobial chemotherapeutics for systemic administration capable of acting against these resistant infectious pathogens. From this point of view, formulation innovations of existing drugs are gaining prominence, while the application of nanotechnologies is a certain alternative for improving/increasing the effect of existing antimicrobial drugs. In all in vitro and in vivo tests, nanomaterials appear to be an effective means of treating and alleviating infections caused by resistant bacteria. Microbial cells are unlikely to develop resistance to nanomaterials because nanomaterials, unlike conventional antibiotics, show toxicity through many mechanisms. It is important to distinguish whether the used material has its own antimicrobial activity (e.g., essential oils, antibiotics, silver compounds, chitosan) or antibacterial activity that is nanospecific, i.e., is achieved at a nanoscale dimension. The intrinsic antimicrobial activity can be potentiated by nanonization; nanoformulations can increase the bioavailability of active substances and modify the route of administration. Some nanoformulations also provide controlled release or targeted biodistribution, which may reduce dose-dependent toxicity and the occurrence of side effects. Increased efficacy of individual drugs at the nanoscale can also be ensured by fixed dose drug combinations or by the encapsulation in antimicrobially active matrices. In addition, many formulations protect drug molecules from degradation. On the other hand, it must be admitted that materials that have acquired their antimicrobial activity at the nanoscale and act on bacterial cells non-selectively (i.e., based on their physical or chemical properties), which limits their primarily systemic applicability. They appear to be very good surface antiseptics and disinfectants, but the non-selective activity caused by their nanosize is a handicap in the fight against systemic infections because of the possibility of their non-selective cytotoxicity (i.e., toxicity against both bacteria and human cells). Therefore, several nanoscale pesticides or packaging films with antimicrobial properties can be found on the market, but real medicaments based on the antibacterial action of nanomaterial or combinations of nanomaterials are still in research and development. A separate chapter preventing their widespread distribution is the stability of these materials both in the organism and after their elimination, which is of course also associated with both cytotoxicity against mammalian cells and non-target organisms and the consequent environmental load. In view of the above-mentioned, it can be stated that the application of nanomaterials for antimicrobial therapy is a promising direction, but the proposed products will still have to be thoroughly investigated before they can be approved for systemic administration to humans.

## Figures and Tables

**Table 1:** Types and composition of discussed nanocarriers (NCs).

Lipid-Based NCs	Micelle-like Structures	Polymer-Based NCs	Metal-Based NCs	Metalloid-Based NCs
liposomes	cyclodextrins	chitosan	silver	selenium
solid-lipid nanosystems	oleylamine	alginate	gold	silica
liquid crystalline NPs	polyethylene glycol	starch	copper	aluminosilica
	polycaprolactone	cellulose	zinc	
		hyaluronic acid	iron	
		pectin	titanium	
		collagen		
		polylactic acid		
		poly(lactic-co-glycolic acid)		
		polyvinyl alcohol		
		polymethacrylate		

## Data Availability

Not applicable.

## Abbreviations

ALG (alginate); AMPs (antimicrobial peptides); BC (bacterial cellulose); CDs (cyclodextrins); CIP (ciprofloxacin); CLI (clindamycin); CNFs (cellulose nanofibers); CS (chitosan); CUR (curcumin); GCS (glycol CS); HA (hyaluronic acid); LEV (levofloxacin); LIN (linezolid); LPHs (lipid polymer hybrids); MDR (multidrug-resistant); MICs (minimum inhibitory concentrations); MRSA (methicillin-resistant *Staphylococcus aureus*); MSSA (methicillin-susceptible *S. aureus*); NCs (nanocarriers); NIR (near infrared); NLCs (nanostructured lipid carriers); NPs (nanoparticles); OFL (ofloxacin); PEG (polyethylene glycol); PGA (poly(γ-glutamic acid)); PCL (poly(ε-caprolactone)); PLA (poly(L-lactic acid); PLGA (poly(D,L-lactic-co-glycolic acid)); PVA (polyvinyl alcohol); RIF (rifampicin); SLNPs (solid lipid nanoparticles); TPP (tripolyphosphate); VAN (vancomycin); VRE (vancomycin-resistant enterococci); VRSA (vancomycin-resistant *S. aureus*).
